# Revamping Parkinson’s disease therapy using PLGA-based drug delivery systems

**DOI:** 10.1038/s41531-025-01081-1

**Published:** 2025-08-20

**Authors:** Jude Majed Lababidi, Hassan Mohamed El-Said Azzazy

**Affiliations:** Department of Chemistry, School of Sciences & Engineering, The American University in Cairo, AUC Avenue, New Cairo, Egypt

**Keywords:** Drug discovery, Molecular biology, Neuroscience, Medical research, Neurology

## Abstract

Parkinson’s Disease (PD) involves degeneration of dopamine-producing neurons, mitochondrial dysfunction, alpha-synuclein aggregation, neuroinflammation, and gut-brain axis disturbances. Despite the availability of pharmacological treatments, these interventions fail to prevent disease progression due to their limited ability to penetrate the blood-brain barrier (BBB) and systemic side effects. Phytochemicals, known for their antioxidant and neuroprotective properties, offer a complementary approach to PD treatment. However, their therapeutic potential is limited by rapid metabolism and poor bioavailability. Several nanoparticles were suggested to enhance the stability and bioavailability of therapeutic agents while enabling controlled release and improved BBB penetration. This review is focused on the use of poly (lactic-co-glycolic acid) (PLGA)-based nanosystem as advanced drug delivery carriers for PD due to its versatility, safety, biodegradability, and extensive studies which evaluated the use of PLGA for drug delivery. It also evaluates their use for encapsulating pharmacological drugs such as dopamine agonists, dopamine precursors, COMT inhibitors, and MAO-B inhibitors, addressing the limitations of conventional therapies. Additionally, the review highlights the utility of PLGA nanoparticles in delivering phytochemicals with neuroprotective effects such as polyphenols, flavonoids, and coumarins to overcome challenges associated with their solubility and stability and ultimately enhance their activities for managing PD.

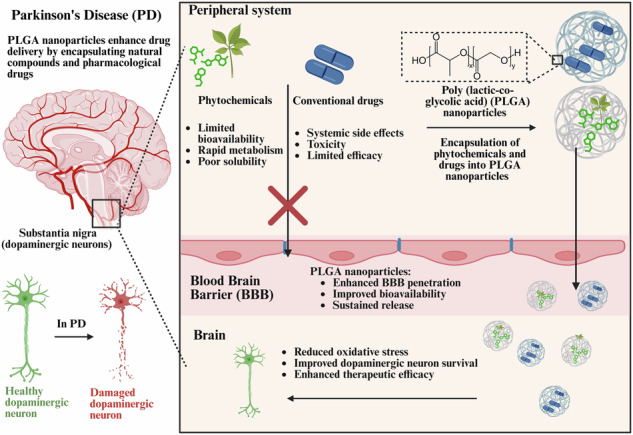

## Introduction

PD is a challenging neurodegenerative disease due to its complex clinical presentation. Over 7–10 million individuals globally are diagnosed with PD^1^. As the 2^nd^ most common neurodegenerative disease after Alzheimer’s disease, PD poses significant clinical and socioeconomic burdens^2^. The pathological features of PD is the selective loss of dopamine producing neurons within the substantia nigra, resulting in a significant decline in striatal dopamine levels^3^. PD is characterized by both motor impairments, such as tremors and rigidity, and non-motor symptoms, including gastrointestinal dysfunction, mood disorders, and cognitive decline^4^. This broad spectrum of manifestations reflects the complexity of its pathophysiology, which involves a combination of dopaminergic neuron loss, oxidative stress, alpha-synuclein aggregation into Lewy bodies, mitochondrial dysfunction, neuroinflammation, and disruptions in the gut-brain axis^5–9^. Furthermore, genetic factors such as mutations in SNCA gene (encodes alpha-synuclein protein) and Parkin (encodes E3 ubiquitin ligase) also contribute to familial forms of the disease^10^.

Conventional pharmaceutical agents, which could provide symptomatic relief, fail to address the complex pathologies of PD^4,11^. Levodopa and dopamine agonists remains the cornerstone of PD therapy due to their ability to cross the BBB and replenish dopamine levels^12^. However, their efficacy diminishes over time, often causing complications including dyskinesias and motor fluctuations^13,14^. Adjunct drugs such as catechol-O-methyltransferase (COMT) and monoamine oxidase-B (MAO-B) inhibitors lack specificity to the central nervous system (CNS), have poor BBB permeability, and result in systemic side effects^15,16^. Furthermore, neuroprotective and disease-modifying agents, including histone deacetylase 6 (HDAC6) inhibitors and metabolic regulators like metformin, show mechanistic promise but face challenges such as poor solubility, rapid clearance, non-specific distribution, and low brain bioavailability^17–20^. This underscores the urgency to advance PD therapeutic approaches.

Natural bioactive products were explored to augment traditional PD drugs. Compounds such as curcumin^21^, resveratrol^22^, and quercetin^23^ were reported to possess neuroprotective, anti-inflammatory, and antioxidant activities that could address the pathological manifestations of PD^24^. However, their clinical translation is impeded by their low bioavailability, poor stability, and inability to cross the BBB^25^.

In recent years, innovative nanocarriers were utilized to enhance the stability, bioavailability, and targeted delivery of therapeutic agents^26^. These platforms could encapsulate drugs and/or natural compounds in protective vehicles, allowing for controlled and sustained release, improved BBB penetration, and reduced systemic toxicity^27^. They also enabled the delivery of poorly soluble compounds with low bioavailability and rapid clearance^28^. Among these nanosytsems, polymer-based carriers, including polycaprolactone (PCL), polyethylene glycol (PEG), and chitosan, PLGA, offered an attractive biodegradable platform for encapsulating both synthetic drugs and bioactive natural compounds^29^. Compared to other polymers, PLGA offers unique properties including high biocompatibility, complete biodegradability into non-toxic lactic and glycolic acids, and suitability for encapsulating both hydrophobic and hydrophilic compounds. These features position PLGA as a superior carrier for CNS drug delivery applications^30^. Additionally, the amenability of PLGA nanosystems to functionalization by ligands (lactoferrin) or polymers (PEG) could facilitate targeted delivery, ensuring efficient delivery of their cargos to target tissues and thus minimizing off-target effects^31^.

This review examines the therapeutic potential of PLGA-based nanocarriers in PD treatment. The pathophysiology of PD, limitations of the current treatment strategies, and the major obstacles of its therapies, including the BBB, progressive neurodegeneration, and challenges with targeting specificity are presented. Findings of studies involving the encapsulation of dopaminergic agents (dopamine, levodopa), dopamine agonists (ropinirole, pramipexole, bromocriptine), enzyme inhibitors (MAO-B and COMT inhibitors), HDAC6 inhibitors, and metabolic regulators (metformin) within PLGA nanoparticles are summarized. The review also discusses the physicochemical characteristics of PLGA nanoparticles loaded with neuroprotective compounds, their in vitro/in vivo performance in terms of BBB permeability, biocompatibility, dopaminergic neuron preservation, and behavioral improvements in PD models.

## Parkinson’s disease

**Fig. 1:**
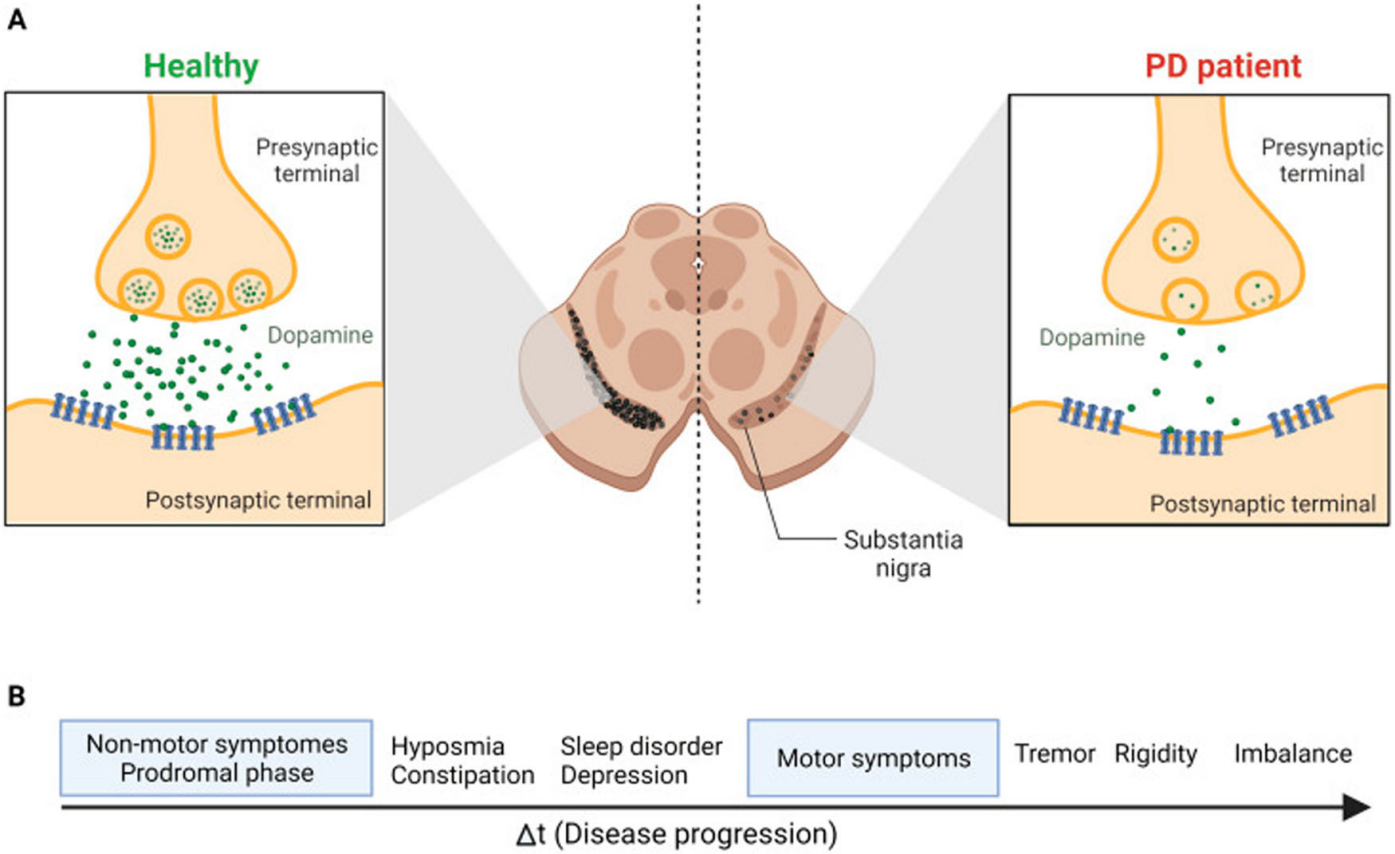
Dopaminergic dysfunction and symptom progression in PD. **A** Synaptic changes in PD. In a healthy individual, dopamine is adequately released from the presynaptic terminal then binds to receptors on the postsynaptic terminal, facilitating normal neural signal transmission. In a PD patient, the degeneration of dopaminergic neurons in the substantia nigra leads to reduced dopamine release, impairing synaptic function. **B** Timeline of PD progression. Early stages (prodromal phase) present with non-motor symptoms such as hyposmia, constipation, sleep disorders, and depression. As the disease progresses, motor symptoms including tremors, rigidity, and postural imbalance become more prominent. Reprinted with permission from ref. ^82^, American Chemical Society, 2022.

PD is characterized by degeneration of dopaminergic neurons, affecting millions of people worldwide and ranking as the second most common condition of its kind after Alzheimer’s disease^32^. PD involves the progressive degeneration of dopamine-producing neurons in the substantia nigra, resulting in reduced dopamine neurotransmitter in the striatum as shown in Fig. 1. This leads to disruption in motor functions, driven by mitochondrial dysfunction, aggregation of alpha-synuclein proteins into Lewy bodies, chronic neuroinflammation, and oxidative stress^33^. Not only that, PD also characterized by a non-motor components as well. Thus, the complexity of these mechanisms underscores the need for an innovative approach to diagnose and treat this disease.

The risk of developing PD increases with age significantly, impacting roughly 1% of individuals over 60 and as many as 4% of those aged 80 or older^34^. Women are ~1.5 times less likely than men to develop PD, suggesting gender-specific risk factors. In the United States, nearly 90,000 new cases are identified annually, and the number has grown by 50% in recent years possibly due to advancements in diagnostic techniques^34^.

### Pathophysiology beyond dopaminergic neuronal loss

#### The alpha-synuclein model

**Fig. 2:**
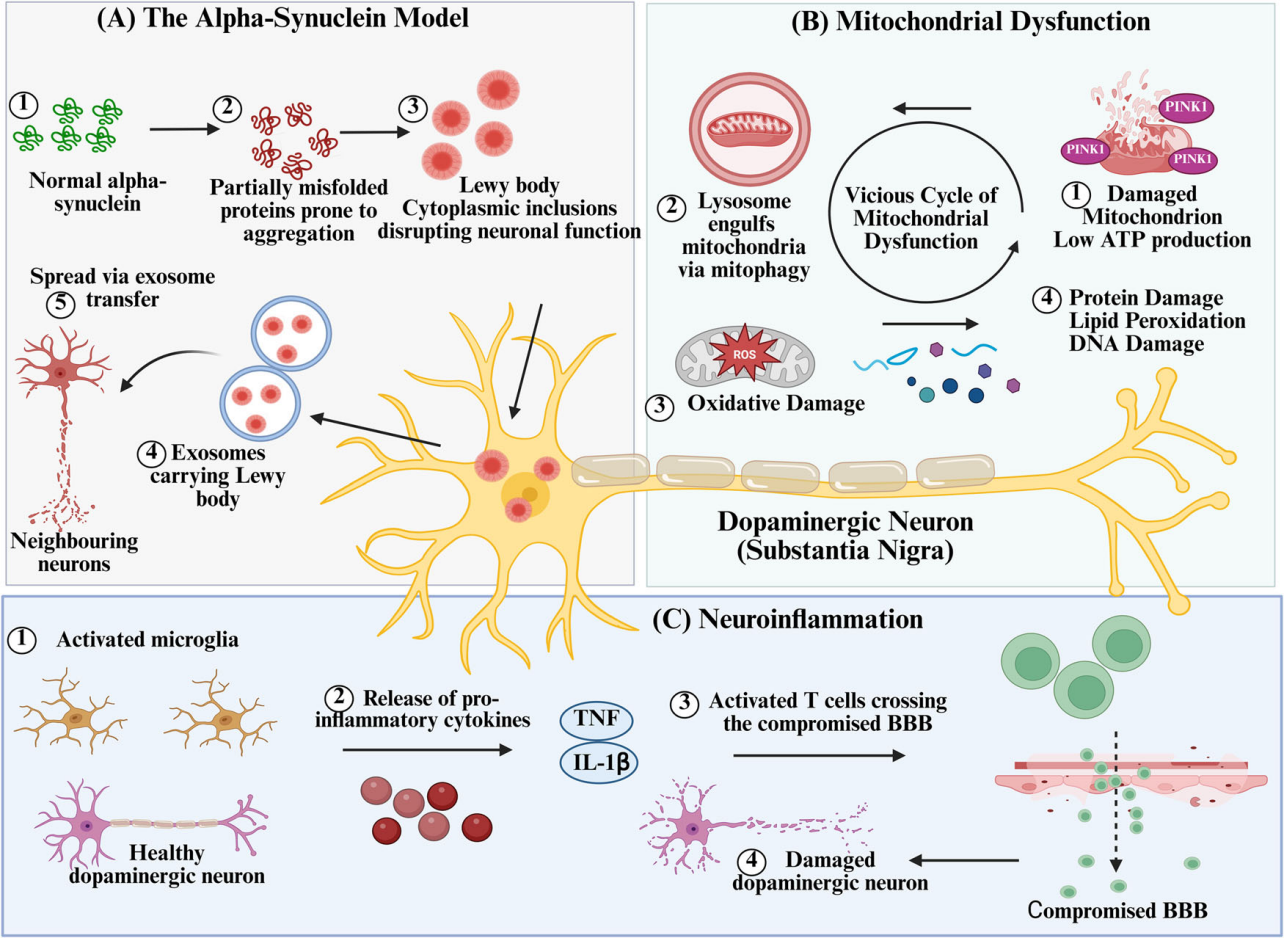
Pathophysiological mechanisms in PD. **A** The alpha-synuclein model shows that misfolded alpha-synuclein aggregates to form Lewy bodies within dopaminergic neurons, disrupting neuronal function. Exosome-mediated spread of misfolded alpha-synuclein to neighboring neurons amplifies aggregation, contributing to disease progression. **B** Mitochondrial dysfunction leads to insufficient ATP production and generates excessive ROS, leading to oxidative damage to proteins, lipids, and DNA. Impaired mitophagy further exacerbates mitochondrial dysfunction, creating a vicious cycle. **C** Neuroinflammation and chronic activation of microglia leads to the release of pro-inflammatory cytokines, such as TNF-α and IL-1β, amplifying neuronal damage. Compromise BBB permits infiltration of activated T cells, intensifying neuroinflammatory responses and contributing to dopaminergic neuron loss.

Alpha-synuclein is a presynaptic neuronal protein whose abnormal aggregation into Lewy bodies leads to disruption in cellular homeostasis and consequently neurodegeneration^7^. The factors initiating the misfolding of alpha-synuclein are complex and multifaceted. Genetic mutations, such as those in the SNCA gene encoding alpha-synuclein, were implicated in familial PD cases^35^. Oxidative stress and exposure to neurotoxins may also induce conformational changes in alpha-synuclein, promoting its aggregation into toxic forms. Post-translational modifications, such as nitration and phosphorylation, further enhance the tendency of alpha-synuclein to misfold and aggregate^7,36^. Studies have shown that alpha-synuclein can be packaged into exosomes, which are then taken up by neighboring cells, leading to the internalization and seeding of alpha-synuclein aggregation in recipient cells (Fig. 2)^37,38^.

#### Mitochondrial dysfunction

Disruption of mitochondrial defense mechanisms is mediated by the Parkin and PTEN-induced kinase 1 (PINK1) genes which are critical for mitophagy, a process that removes dysfunctional mitochondria from cells. In normal cases, PINK1 is degraded by healthy mitochondria, but when mitochondria become dysfunctional or damaged, PINK1 accumulates on their outer membranes, recruiting Parkin to mark the defective organelles for autophagic degradation. Mutations in Parkin or PINK1 impair these proteins, leading to the accumulation of damaged mitochondria, reduced energy production, and elevated levels of reactive oxygen species (ROS)^39^. The resulting oxidative stress damages cellular proteins, lipids, and DNA, creating a vicious cycle that exacerbates neuronal degeneration (Fig. 2)^40^.

#### Neuroinflammation and the immune system

Microglia mediate the immunomodulatory processes in the brain and play a crucial role in the neuroinflammation in PD, leading to chronic activation and releasing of pro-inflammatory cytokines such as tumor necrosis factor-alpha (TNF-α) and interleukin-1 beta (IL-1β), which further increase the damage in neurons and promote disease progression^41^. This persistent neuroinflammation is further intensified by interactions with the peripheral immune system. Activated T cells can infiltrate the brain by crossing a compromised BBB, amplifying inflammatory responses and contributing to dopaminergic neuron loss^42^. Additionally, increased intestinal permeability, called leaky gut, allows gut-derived pro-inflammatory molecules and microbial metabolites to enter systemic circulation, enhancing systemic inflammation and influencing central neuroinflammatory processes (Fig. 2)^43^.

### Non-motor symptoms: the hidden burden

#### Gastrointestinal dysfunction and the gut-brain axis

PD may originate in the gut before manifesting in the central nervous system (CNS) through the formation of alpha-synuclein aggregates within the visceral nervous system. Additionally, analyses of the gut microbiota in PD patients revealed dysbiosis characterized by a reduction of bacteria producing short-chain fatty acid which are crucial for maintaining the barrier integrity of the intestines^44,45^. These findings support the hypothesis that gastrointestinal dysfunction could precede motor symptoms in PD. Additionally, the bidirectional interaction between CNS and peripheral immunity highlights the role of gut-brain axis dysfunction in PD. Disruptions in gut microbiota composition may exacerbate inflammation through the vagal nerve and the weakened BBB, establishing a feedback loop which accelerates disease progression^46^. Consequently, strategies like dietary modifications, administration of probiotics, and microbiome-targeted therapies are being explored to restore gut microbial balance and mitigate disease progression^44,47^.

#### Cognitive decline and neuropsychiatric symptoms

The degeneration of dopaminergic neurons in the basal ganglia disrupts executive functions, leading to difficulties in planning, problem-solving, and multitasking. Additionally, the accumulation of cortical Lewy bodies impairs attention and visuospatial abilities, contributing to challenges in navigation and perception^48^. Beyond cognitive deficits, PD patients frequently experience neuropsychiatric symptoms such as depression and anxiety, which result from widespread neurotransmitter dysregulation, including alterations in serotonin and norepinephrine levels. These neuropsychiatric manifestations are often under-recognized and under-treated, yet they negatively affect daily functioning and overall well-being of PD patients^48–50^.

### Current treatment modalities and limitations

Pharmacological treatment remains the primary current approach to managing PD, with levodopa considered the gold standard for alleviating motor symptoms^51^. Levodopa is a precursor to dopamine that crosses the BBB to be metabolized and converted into dopamine, mitigating some motor dysfunctions such as rigidity, bradykinesia, and tremors^12,51^. However, long-term use of levodopa often leads to side effects, including fluctuations in motor movements characterized by “on” and “off” periods, where the effectiveness of levodopa reported to vary throughout the day. This challenge arises because levodopa undergoes extensive peripheral metabolism, reducing the amount of drug that reaches the brain^52^. Consequently, higher doses are required, increasing the risk of systemic side effects like nausea, hypotension, and dyskinesias, and still failing to achieve targeted delivery to the affected brain regions. To address these limitations, adjunctive therapies are commonly used. Dopamine agonists, including rotigotine, ropinirole, and pramipexole directly stimulate dopamine receptors and can delay the need for levodopa in early-stage PD^53,54^. However, their variable BBB permeability and non-selective action often result in side effects such as neuropsychiatric issues and impulse control disorders^55–57^.

Another class of PD drugs includes the monoamine oxidase-B (MAO-B) inhibitors like rasagiline and selegiline^58^. They inhibit the breakdown of dopamine, and therefore enhance and prolong its action in the brain. These agents are particularly useful as add-on therapies but may also suffer from incomplete CNS penetration and systemic side effects such as hypertension and insomnia due to their off-target activity. Other pharmacological options include catechol-O-methyltransferase (COMT) inhibitors like entacapone and opicapone, which extend the half-life of levodopa, do not cross the BBB at all and primarily affect peripheral metabolism, providing no direct action in the brain^59^. This peripheral-only action limits their efficacy and contributes to gastrointestinal and hepatic side effects.

Furthermore, anticholinergic medications, such as trihexyphenidyl, are used also in PD management but struggle to effectively cross the BBB and often exhibit non-specific binding, leading to undesirable peripheral effects such as dry mouth and cognitive impairment, particularly in elderly patients. Amantadine, an N-methyl-D-aspartate (NMDA) receptor antagonist, may be used for managing dyskinesias and offers mild benefits in reducing motor symptoms. However, it has limited CNS bioavailability and lacks specificity, contributing to short-lived efficacy and side effects including confusion and hallucinations^60,61^. Another class of drugs has been explored for PD management is histone deacetylase 6 (HDAC6) inhibitors such as such as CAY10603 and Tubastatin A which enhance the α-synuclein aggregates clearance to mitigate neurodegeneration^17,62^. Their mechanism involves restoring the acetylation of α-tubulin in neurons, which stabilizes microtubules and enhances mitochondrial transport^63^. Additionally, HDAC6 inhibitors activate redox-regulating proteins like peroxiredoxins (reduce ROS) and support the function of the autophagy and ubiquitin-proteasome system (UPS) (both are impaired in PD patients)^62^. These effects help reduce oxidative stress and improve protein clearance capacity in affected neurons^64^. So, these agents target the underlying pathophysiology of PD, unlike traditional symptomatic treatments like MAO-B inhibitors, dopamine agonists, or COMT inhibitors. However, HDAC6 inhibitors are poorly soluble in water which restricts their bioavailability and BBB permeability. Additionally, their specificity remains a concern as they could inhibit other HDAC isoforms leading to unwanted side effects^65,66^. Finally, metformin belongs to biguanide group commonly used in type II diabetes mellitus as it lowers blood glucose level via activation of AMP-activated protein kinase (AMPK). Specifically, metformin recently gained attention for its neuroprotective effect in PD. The activation of AMPK promotes the clearance of misfolded proteins such as α-synuclein and supports the function of mitochondria by stimulating peroxisome proliferator-activated receptor gamma coactivator-1 alpha (PGC-1α, a regulator of biogenesis of mitochondria). Additionally, metformin upregulates nuclear respiratory factors like Nrf2, reducing the oxidative stress. It also relieves neuroinflammation by preventing the release of pro-inflammatory cytokines and microglial activation^18,67^. While metformin can cross the BBB, it has poor solubility and its brain concentration is low to induce sufficient neuroprotective action^19,67^. This highlights the need for strategies to overcome the restrictive nature of the BBB and the lack of drug targeting specificity which hinder delivery of adequate drug concentrations in the affected brain regions.

## Obstacles to Parkinson’s disease therapy

### The blood-brain barrier

The BBB maintains homeostasis by controlling the exchange of substances between the bloodstream and the CNS. BBB also provides protection to neural tissues against toxins and pathogens which may be present in circulation. The BBB achieves this selectivity through the endothelial cells which are connected by tight junctions, supported by the basement membrane, astrocytic end-feet, and pericytes^68^. It employs specialized transport systems to allow essential nutrients and metabolites to cross into the brain^69^. For example, amino acids are carried by the large neutral amino acid transporter 1 (LAT1), glucose is transported *via* the glucose transporter 1 (GLUT1), and small peptides utilize the peptide transporter 1 (PEPT1). Moreover, active efflux transporters, such as breast cancer resistance protein (BCRP), P-glycoprotein (P-gp), and multidrug resistance-associated proteins (MRPs), expel many therapeutic agents from the endothelial cells back into the bloodstream, significantly reducing their CNS infiltration^69,70^. Additionally, the BBB exhibits regional heterogeneity in permeability, which can vary across different brain areas and pathological conditions. In PD, neuroinflammatory processes may further compromise BBB integrity, leading to localized disruptions and uneven permeability. This variability makes it challenging to achieve consistent drug delivery across all affected brain regions. Systemic administration of therapeutics also results in off-target effects, as only a small fraction of the administered dose crosses the BBB, with the remainder accumulating and affecting peripheral tissues^71,72^.

### Progressive neurodegeneration and neuroinflammation

The combination of progressive neurodegeneration and chronic neuroinflammation in PD significantly reduces the effectiveness of PD therapies. As the dopamine-producing neurons in the substantia nigra are lost, the availability of therapeutic targets diminishes, while receptor desensitization and downregulation further impair drug effectiveness. Neuroinflammation disrupts the neuronal microenvironment, interfering with drug action and accelerating the aggregation of alpha-synuclein^73^. This pathological processes extend beyond the dopaminergic system, rendering therapies focused on motor symptoms ineffective for managing non-motor complications like cognitive decline and mood disturbances. Additionally, inflammation-induced oxidative stress can degrade drugs^70,74,75^.

### Late diagnosis and limited therapeutic effectiveness

PD is often diagnosed only after substantial neurodegeneration has occurred where ~60–80% of dopamine-producing neurons have been lost. Thus, the remaining neurons fail to produce adequate amount of dopamine. Additionally, available pharmacological treatments do not target the root causes of the disease or slow down its progression^76^.

### Lack of targeting specificity

Current treatments are not designed to specifically target diseased neurons. This limits efficacy and increases the risk of off-target effects, including neuropsychiatric side effects and motor complications^77^. Furthermore, uncertainty in accounting for individual patient variability, such as genetic predispositions, disease stage, or coexisting conditions, makes available drugs less effective across diverse patient populations^78^.

## Dopamine restoration in Parkinson’s patient

Restoring normal dopamine levels is key to managing PD, but there are significant challenges with current treatments. Dopamine cannot cross BBB on its own. This is why precursors like levodopa are used which can cross the BBB and are metabolized and converted into dopamine in the brain. However, when dopaminergic neurons (the cells responsible for this conversion) are stressed in PD, their ability to perform this conversion becomes impaired. One proposed solution is to administer dopamine directly to the brain, bypassing the need for its enzymatic conversion from levodopa. While this seems promising, it has significant challenges. Dopamine is highly prone to oxidative stress when administered outside the BBB because dopamine is broken down by an enzyme MAO, producing ROS. Subsequently, ROS harm cells by damaging important components like DNA, lipids, and proteins, which worsens the condition of already compromised neurons. These issues highlight the need for advanced strategies to safely deliver dopamine to the brain while protecting it from oxidative damage^79^. Drug delivery using various nanosystems, such as liposomes^80^, dendrimers^81^, and polymeric nanoparticles^82^, offer promising advantages. These systems can protect dopamine from degradation, enable targeted delivery to damaged areas of the brain, and provide a controlled, sustained release of dopamine.

## The potential of natural products in PD treatment and their challenges

The significant limitations of current PD pharmacological therapies have sparked growing interest in natural products as alternative or adjunctive therapies. Natural compounds such as quercetin^83^, resveratrol^22^, and curcumin^21,84^ have shown great potential in ameliorating the underlying causes of PD due to their impressive neuroprotective, anti-inflammatory, and antioxidant properties. For instance, Zhang et al. suggested that curcumin plays a key role in scavenging ROS and thus reduces oxidative stress and neuronal damage^85^. Lofrumento et al. found that resveratrol decreases inflammation in neurons by controlling the activation of microglial cells and modulating the pro-inflammatory cytokines^86^. Quercetin, on the other hand, has shown promise in inhibiting alpha-synuclein aggregation and protecting mitochondrial function^87^. Despite these promising therapeutic properties, the clinical utility of natural products in PD remains limited due to several challenges. First, many natural compounds have poor bioavailability as they are poorly absorbed and metabolized rapidly, leading to insufficient therapeutic concentrations at the target site. Second, their ability to cross the BBB is restricted. Third, these compounds often exhibit poor stability, degrading quickly under physiological conditions, which reduces their efficacy. Together, these shortcomings prevent natural products from reaching the affected brain regions in concentrations sufficient to exert meaningful therapeutic effects^88,89^. To overcome these limitations, advanced drug delivery systems, particularly nanotechnology-based approaches, have emerged as promising solutions. These nanocarriers offer several advantages, including protecting encapsulated natural products from enzymatic degradation, improving their solubility, enabling sustained and controlled release of the active compounds over time. Furthermore, by functionalizing nanocarriers with specific ligands, it is possible to facilitate their passage across the BBB and achieve targeted delivery to the degenerated brain regions.

## PLGA nanocarriers for better drug delivery in PD

Encapsulating pharmacological drugs and natural compounds within nanosystems is reported to significantly enhance the therapeutic potential of treatments for various diseases^90^. Nanosystems improve solubility, stability, and bioavailability of their drug cargos making them especially valuable for treating cancers^91^, cardiovascular diseases^92^, and inflammatory conditions^93^. Specialized nanosystems have shown unique advantages in addressing neurological diseases due to their ability to overcome the BBB, the major obstacle for delivery of therapeutic agents into the brain. One of the most promising aspects of nanosystems in treating neurological disorders lies in their small size and capacity to traverse the BBB^94^. This attribute makes them uniquely suitable for targeting the CNS, where conventional drugs often fail. Among the various polymeric materials employed in nanosystems such as polycaprolactone (PCL), chitosan, polyethylene glycol (PEG), and polyvinyl alcohol (PVA), PLGA stands out due to its unique properties. PLGA exhibits high encapsulation efficiency for both hydrophobic and hydrophilic compounds unlike chitosan, which is more suitable for hydrophilic drugs^30,95^. While PEG is non-biodegradable^96^, PLGA is metabolized into non-toxic byproducts (lactic and glycolic acids), making it a biodegradable and a biocompatible polymer^97^. Additionally, PLGA offers customizable release profiles when compared to PCL that has very slow degradation rate, limiting its applicability in controlled drug release^98^. Functionalization of PLGA nanoparticles could further enhance BBB targeting making them advantageous for delivering therapeutic agents to manage PD and other CNS disorders^99^. In this context, PLGA nanocarriers have emerged as an effective platform for delivering pharmacological agents and natural compounds including essential oils^100^, plant extracts^101^, and bioactive molecules^102,103^. This review delves into the applications and benefits of PLGA nanocarriers for drug delivery in neurological disorders, focusing particularly on PD. Delivering pharmacological drugs and natural compounds using PLGA nanocarriers, examining their chemical composition and safety profiles, and analyzing their advantages for PD treatment are covered.

### Poly lactic-co-glycolic acid (PLGA) nanoparticles

**Fig. 3:**
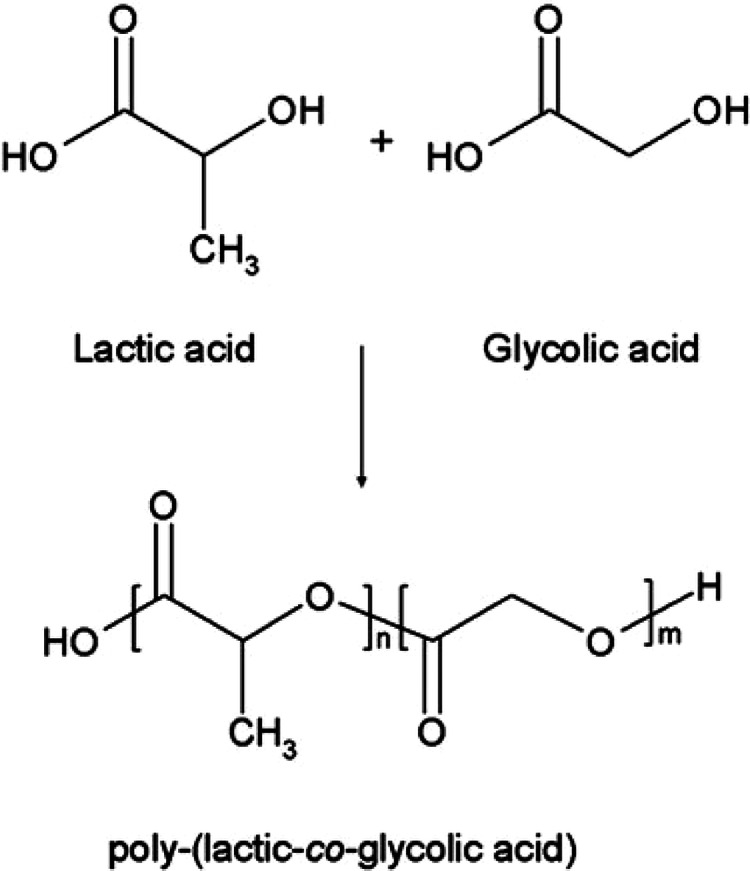
Chemical structure of PLGA. Reprinted with permission from ref. ^106^, MDPI,2014.

From its original use in surgical sutures to its current use in advanced drug delivery systems, PLGA is a prominent biodegradable polymer whose development reflects decades of innovation in biomaterials research. The roots of PLGA lie in the exploration of polylactic acid (PLA) and polyglycolic acid (PGA) as biodegradable polymers. In the 1960s, PGA sutures, marketed under the brand name Dexon^TM^, were introduced as the first synthetic absorbable sutures^104^. Soon after, Vicryl sutures, a combination of PLA and PGA, were developed in the 1970s to address the stiffness of PGA by improving flexibility with lactic acid^105^. This success spurred research into copolymers (Fig. 3) leading to the discovery of PLGA as a material with customizable properties through varying the lactic-to-glycolic acid ratio^105,106^. The variability in molar ratios influences the physicochemical properties of PLGA such as its degradation rate and surface wettability. For example, higher lactic acid content delays degradation and increase hydrophobicity, while increased glycolic acid proportion accelerates hydrolysis and enhances hydrophilicity^107^. At room temperature, PLGA remains in a rigid state since its glass transition temperature lies within (20–51.5 °C)^108^. PLGA is insoluble in aqueous media, but it can dissolve in organic solvents, such as acetone and dimethylformamide (DMF)^109^. The terminal carboxylic groups of PLGA chains impart its surface negatively charged, which can be modified through functionalization to facilitate targeted drug delivery^110^. Additionally, the hydrolysis of ester linkages degrades PLGA into its monomers (glycolic and lactic acid), which could be metabolized and eliminated by the body, reflecting its biocompatibility^97^. Regarding safety, PLGA showed limited cytotoxicity in various in vitro *and* in vivo studies^111^. However, the bulk degradation of PLGA may cause localized inflammation due to acidic products formed, especially in poorly buffered systems^97^. To mitigate excessive acidic byproducts, block co-polymers and stabilizers were included in PLGA formulations^112^. Furthermore, PLGA gained food and drug administration (FDA) approval in the 1980s, marking its entry into advanced medical applications^111^. In 1989, Lupron Depot^TM^, a controlled-release formulation for prostate cancer, became one of the first commercial products to use PLGA microspheres^113,114^. The versatility of PLGA allowed for its use in other sustained-release products, including Risperdal Consta, approved in the early 2000s for schizophrenia treatment^115^. Interestingly, PLGA found a new role in the 21st century as a key material for drug-loaded nanoparticles. Its ability to encapsulate a variety of therapeutic agents and natural compounds enhanced its appeal^115^. Functionalized PLGA nanoparticles, modified with ligands or peptides, demonstrated the ability to cross the BBB, enabling their use in neurological disorders like PD^116,117^. Today, several research studies report their advantage in delivering both natural compounds and pharmacological drugs for PD treatment.

### Mechanisms of PLGA-based nanoparticles transport across the BBB

The transport of PLGA-based nanoparticles across the BBB is mediated by active or passive transporters^118^. PLGA-based nanoparticles cross the BBB passively by adsorptive-mediated transcytosis (AMT), which is driven by electrostatic interactions between the endothelial surface of the BBB (negatively charged) and PLGA-based nanoparticles (positively charged). While PLGA nanoparticles carry a negative surface charge, surface modification strategies, such as coating with cationic surfactants or chitosan can impart positive surface charge. This electrostatic interaction facilitates non-specific endocytosis and subsequent transcytosis of the nanoparticles across the BBB. AMT lacks receptor specificity, which increases the off-target distribution^118,119^. PLGA-based nanoparticles can cross the BBB by active processes namely receptor-mediated transcytosis (RMT) and carrier-mediated transport (CMT). RMT is restricted to regions of high receptor density such as the microvascular endothelium of the cerebral cortex and basal ganglia. This pathway involves functionalizing PLGA nanoparticles with ligands that specifically bind to receptors highly expressed on the endothelial cells of the brain, including transferrin, lactoferrin, insulin, and low-density lipoprotein receptors. Upon ligand binding, the nanoparticle-receptor complex is internalized via clathrin-coated vesicles to be transported across the BBB and subsequently released by exocytosis into the brain parenchyma^119,120^. On The other hand, CMT has limited transport capacity compared to RMT due to substrate specificity like glucose transporter (GLUT1) or large neutral amino acid transporter (LAT1). PLGA-based nanoparticles that are conjugated with substrates of carrier transporters can pass through the BBB. These transporters are abundantly expressed on the luminal side of endothelial cells in the BBB^120^. Some studies also suggest that tight junction modulation by surfactants or targeting peptides can enhance BBB permeability transiently, though safety concerns remain^69^. Thus, understanding these mechanisms is crucial for enhancing the delivery of PLGA nanoparticles across the BBB to minimize systemic toxicity

### Pharmacological drugs loaded PLGA nanoparticles for PD treatment

Several studies demonstrated the effectiveness of PLGA nanosystem in encapsulating pharmacological treatments, thereby improving bioavailability, enhancing BBB penetration, and providing sustained drug release. Table 1. summarizes the characterization techniques, delivery systems, and key outcomes of various drugs encapsulated in PLGA nanoparticles for PD treatment

**Table 1:** Characteristics of selected nanosystems for delivery of PD drugs

Classification/Drug	Delivery system	Characterization of prepared nanoparticles	Cell line/animal model (route of administration)	Key outcomes	Ref
Dopamine	Dopamine-loaded albumin/PLGA nanoparticles	Particle size: 266 ± 8 nm. PDI: 0.178 ± 0.028. Zetapotential: +29.7 ± 5.17 mV. EE: 36.3 ± 5.4%. DL: 18.7 ± 2.5.	6-OHDA PD mice model (Intraperitoneal)	-Crossed the BBB efficiently to deliver dopamine to the brain parenchyma, -Improved motor coordination, balance, and sensorimotor function -Reduced apomorphine-induced rotational behavior, and restored dopamine at the nigrostriatal pathway.	^121^
Dopamine	Dopamine-loaded PLGA (Poloxamer 188-coated)	Particle size: 198.1 ± 3.4 nm. PDI: 0.03 ± 0.01. Zetapotential: −29.1 ± 1.7 mV. Dopamine content: 4.1 ± 0.4 µg/mg.	Advanced hiPSC-derived BBB model, bEnd.3, LUHMES cells (In vitro)	-Successful transportation of 0.94% of dopamine across the BBB model within 4 h. -High uptake was observed in LUHMES dopaminergic neurons (64.9%) and bEnd.3 cells (89.2%), with no cytotoxic effects.	^122^
Dopamine	Lactoferrin and Borneol Co-Modified PEG-PLGA	Particle size: 175.3 ± 9.6 nm. PDI: 0.129 ± 0.011. Zetapotential: −15.7 ± 0.86 mV. EE: 25.43 ± 5.32%. Release profile: 70% in 72 h.	SH-SY5Y, Human bronchial epithelial cells (16HBE) cells; 6-OHDA-induced rat model (Intranasal)	-Improved dopamine delivery to the brain, with significantly higher brain/blood ratios and reduced contralateral rotations in a PD rat model. -Enhanced cellular uptake and low cytotoxicity were observed.	^151^
Dopamine precursor/Levodopa	Wheat germ agglutinin -levodopa PLGA	Particle size: 711.6 ± 339.8 nm. Zetapotential: −23.2 ± 2.38 mV. PDI: 0.650 ± 0.178. EE: 14.75%. Release (12–18 h): 80% of levodopa DSC, FTIR.	–	–	^152^
Dopamine precursor/Levodopa	Levodopa-PLGA	Particle size: 553 ± 52 nm. PDI: 0.522. Zetapotential: +46.2 ± 2.3 mV. EE: 82.38 ± 1.63%. FTIR, XRD.	Male Wistar rats (Intranasal administration)	-Facilitated sustained release -Increased nasal residence time compared to free levodopa solution.	^153^
Dopamine precursor/Levodopa	Levodopa-PLGA	Particle size: 46 nm. PDI: 0.1. Zetapotential: +16 mV.	Reserpine-induced PD rat model (subcutaneous administration at the neck)	-Improved brain delivery *via* lymphatic vasculature -High dopamine levels in corpus striatum (~1.3 ng/mL) -Improved movement disorders in PD rat model (pole test, rotarod, hanging tests) -Reduced oxidative stress -Biocompatibility confirmed with no toxicity to major organs	^154^
Dopamine precursor/ Levodopa and Carbidopa	Levodopa-carbidopa-PLGA	Particle size: 14 ± 1.8 μm. PDI: 0.12. EE: levodopa was >97% and >68% for carbidopa.	Simulated Gastric Fluid and Intestinal Fluid (In vitro oral administration simulation)	-Sustained release of both drugs with >80% release in 5 h and >90% release in 24 h -Follows anomalous transport mechanism (diffusion and degradation)	^123^
Dopamine precursor/ Levodopa and Benserazide	Levodopa-benserazide-PLGA	Particle size: 500 nm. 100% release of the drugs over 2 weeks.	6-OHDA-lesioned PD rat model (Subcutaneous injection)	-Sustained release of levodopa-benserazide for ~2 weeks -Prevented levodopa-induced dyskinesia -Overexpression of β-Arrestin2 enhanced anti-dyskinetic effects -Reduced pulsatile effects of levodopa	^124^
Dopamine precursor/Levodopa and Carbidopa, COMT inhibitor/Entacapone	Levodopa-carbidopa-entacapone-PLGA	Particle size: 4.6 ± 0.4 μm. Coefficient of variance: 8.6%, indicating a narrow size distribution. EE: 100% for levodopa, carbidopa, and entacapone. DSC, AFM, XRD, NMR, Raman Spectroscopy.	Rotenone-induced PD rat model (Oral administration)	-Simultaneous sustained release of three drugs for up to 24 h -Improved therapeutic efficacy compared to standard formulations -Increased drug bioavailability (14-fold higher for levodopa) -Reduced dosing frequency (once daily) - Motor function restoration	^155^
Dopamine agonist/Pramipexole	Pramipexole-Au nanospheres-PLGA	Particle size: 24 µm EE: 51.71 ± 0.54% for pramipexole and 65.15 ± 2.30% for Au nanospheres. DSC	C2C12 myotubes, RAW macrophages, PC12 model neurons, 3T3 fibroblasts for cytotoxicity studies, MPTP-induced PD mouse model (Intramuscular injection, near-infrared laser irradiation)	-Reduced dosing frequency (every 2 weeks) -Enhanced pharmacokinetics: higher plasma concentration and AUC -Improved recovery of dopaminergic neurons and motor coordination -Cytotoxicity tests showed biocompatibility of the formula	^125^
Dopamine agonist/Pramipexole	Pramipexol-PLGA with Pluronic F68	Particle size: 195 nm. Zetapotential: 34.8 mV. EE: 86.21% Release of 95.2% of the drug in 24 h. FTIR, DSC.	SH-SY5Y cell line (MTT Assay)	-Higher cell viability compared to free pure drug	^156^
Dopamine agonist/ Ropinirole	Ropinirole-PLGA	Particle size: 196.4 ± 8.68 nm. PDI: 0.059 ± 0.037. Zetapotential: −10.35 ± 0.81 mV.EE: 83.30 ± 0.61%. 24-h release profile: 61.42%. DSC, FTIR.	Wistar Rats (IV injection via tail vein)	-Superior BBB penetration, better brain targeting, and sustained drug release	^157^
Dopamine agonist/ Ropinirole	Ropinirole-Chitosan-PLGA	Particle size: 468 ± 40.0 nm. PDI: 0.29 Zetapotential: +54.4 ± 2.6 mV. LD: 5.7 ± 2.5%. DSC.	PBMCs, RAW 264.7 cells (in vitro); Sheep nasal mucosa (ex vivo)	-3.22-fold enhanced ropinirole permeability across nasal mucosa -Mucoadhesion increased with Chitosan presence. -No significant ROS production or hemolysis at therapeutic concentrations. -Toxic effects are dose-dependent -Histopathology confirmed no significant damage to nasal epithelium.	^126^
Dopamine agonist/ Ropinirole	Ropinirole-PLGA-PVA	For the optimal formula: Particle size: 224.3 ± 8.90 nm PDI: 0.074 ± 0.04. EE: 78.65 ± 0.64%. DL: 1.31 ± 0.011%. Release profile: 79.46 ± 1.08% after 48 h. FTIR, DSC.	Wistar Rats (Intravenous Tail Vein Injection)	-Sustained in vitro release (up to 79.46% over 48 h), following Higuchi diffusion release model. -Effective crossing of the BBB in vivo with significant drug levels in the brain. -Stable formulation for 3 months at 3-5 ± 2 °C.	^158^
Dopamine agonist/ Rotigotine	Lactoferrin-Rotigotine-PLGA	Particle size: 118.4 ± 12.4 nm. Zetapotential: −21.94 ± 2.83 mV. EE: 88.07 ± 6.01%. DL: 8.01 ± 0.05%.	Sprague Dawley rats, 6-OHDA model of PD (Intranasal)	-Enhanced brain-specific delivery -Selective targeting to the striatum. -Superior pharmacodynamic and neuroprotective effects. -Significant reduction in contralateral rotations and dopaminergic neurodegeneration. -Effective targeting *via* lactoferrin receptor-mediated transport.	^159^
Dopamine agonist/ Rotigotine	Lactoferrin-Rotigotine-PLGA	Particle size: 122 ± 19.3 nm PDI: 0.194 ± 0.023 Zetapotential: −21.28 ± 2.15 mV. EE: 92.57 ± 9.41%. Release profile: 77.8% ± 7.0% after 48 h.	16HBE and human neuroblastoma cells (SH-SY5Y); Kunming mice (Intranasal administration)	-Enhanced cellular uptake -Slow and sustained release of rotigotine over 48 h. -Higher brain-specific targeting of rotigotine, particularly to the striatum -Significant increase in rotigotine concentrations in brain regions -Reduced cytotoxicity of rotigotine encapsulated in PLGA	^117^
Dopamine agonist/ Bromocriptine mesylate	LMWP/lactoferrin co-modified PLGA	Particle size: 248.53 ± 16.25 nm. PDI: 0.274. Zeta potential: –2.63 ± 0.74 mV. Spherical shape (TEM). EE%: 73.30 ± 0.74%. DL%: 10.11 ± 0.10%.	16HBE14o– and BCECs. Haloperidol-induced PD mice model in Swiss albino mice (Intranasal)	- Enhanced cellular uptake across nasal and brain barriers (6.5-fold that of unmodified nanoparticles) - Improved behavioral outcomes: reduced catalepsy and akinesia and increased swimming time and grip strength. - Increase in SOD and GSH decrease in MDA levels. - Restored neuronal morphology. - No toxicity or inflammation observed in nasal mucosa.	^127^
MAO-B inhibitor/ Rasagiline	Rasagiline-PLGA	Particle size: 63.681 μm. EE: 89.88%. DL: 30.12%. Prolonged release for 60 days.	6-OHDA-lesioned Sprague–Dawley rats (Intramuscular injection)	-Sustained release for 60 days -Prolonged plasma drug levels over 32 days. -High-dose (30 mg/kg) was most effective in reducing apomorphine-induced rotational behavior and increasing dopamine levels in the lesioned striatum.	^160^
MAO-B inhibitor/ Selegiline	Selegilin-PLGA embedded ethylene vinyl acetate transdermal film	Particle size: 286.1 ± 4.23 nm. PDI: 0.461 ± 0.93. Zetapotential: −29.11 mV.	Reserpine-induced PD in male Wistar rats (Transdermal application)	-Dopamine levels in brain tissue increased significantly (102.31 ± 7.51 ng/g) while MAO-B levels decreased after transdermal film application. -Improvement in cataleptic behavior and brain targeting efficiency -Minimal skin irritation observed after 72 h.	^128^
COMT inhibitor/Tolcapone	Tolcapone -PEGylated PLGA	Particle size: 148 nm. PDI: <0.200. EE: of 47.0%. DL: 5.7%. Release profile: 46% after 24 h.	HepG2 cells (in glucose and galactose-supplemented media for cytotoxicity)	-Cytotoxicity was reduced in HepG2 cells with decreased ROS production and counteracted ATP depletion. -COMT inhibition was achieved at the same level as free tolcapone. -Overcome hepatotoxicity while maintaining therapeutic efficacy.	^129^
COMT inhibitor/Entacapone	Entacapone- PEGylated PLGA	Particle size: 186.0 ± 4.0 nm. PDI: <0.3. EE: 13.1 ± 0.2%. DL: 0.7 ± 0.1%.	SH-SY5Y, HepG2, Caco-2 cell lines	-Improved entacapone permeability in Caco-2 and Caco-2/HT29-MTX coculture models compared to free entacapone. -Low cytotoxicity -COMT inhibition retained at similar levels when compared to free entacapone.	^130^
HDAC6 inhibitor/CAY10603	Lactoferrin-conjugate- CAY10603- loaded PLGA	Particle size: 99.7 ± 9.4 nm by TEM. PDI: 0.09. Zeta potential: −49.2 ± 2.9 mV, EE%: 20.57 ± 1.21%. DL%: 0.69 ± 0.06%. Release over 192 h.	SH-SY5Y, hBMEC cell lines. -Methamphetamine induced PD model in female C57BL/6J mice (Intravenous injection)	-Enhanced BBB penetration and brain accumulation. -3-fold ROS reduction. -11.9-fold increase in acetylated α-tubulin. -3.3-fold reduction in α-synuclein. -Improved DA and DOPAC levels. -Improved behavioral performance. -Reduced astrocyte and microglial activation -Good biocompatibility and minimal hemolysis	^20^
Metformin/AMPK activator)	with BV2 microglial membrane-Metformin-PLGA	TEM: core-shell structure. Particle size: 252.2 nm (DLS). PDI: 0.228. Zeta potential: −20 mV. EE%: 10.76%. DL%: 3.98%.	SH-SY5Y and BV2 cell lines. MPTP-induced PD mouse model in male C57BL/6 mice (intranasal)	-Reduced ROS and iNOS, increased CD206 -Enhanced autophagy (increase of p62) -Restored MMP (JC-1) -Decreased TNF-α, IL-6, GFAP -Increased IL-4, IL-10 -Improved motor function -Reduced α-syn aggregation downregulated VDAC1 and NOX4 -Modulation of inflammation and mitochondrial genes	^19^

*16HBE* human bronchial epithelial cells, *6-OHDA* 6-Hydroxydopamine, *BBB* blood-brain barrier, *Caco-2* human colorectal adenocarcinoma, *Caco-2/HT29-MTX* Co-culture of human colorectal adenocarcinoma and mucin-producing HT29-MTX cells, *COMT* catechol-O-methyltransferase, *DL* drug loading capacity, *EE* encapsulation efficiency, *DSC* differential scanning calorimetry, *FTIR* Fourier transform infrared spectroscopy, *hiPSC* human induced pluripotent stem cells, *HepG2* human hepatocellular carcinoma, *IV* intravenous, *MAO-B* monoamine oxidase B, *MPTP* 1-methyl-4-phenyl-1,2,3,6-tetrahydropyridine, *MMP* mitochondrial membrane potential, *PBMCs* peripheral blood mononuclear cells, *PDI* polydispersity index, *PVA* polyvinyl alcohol, *RAW 264.7* mouse macrophage, *ROS* reactive oxygen species, *XRD* X-ray diffraction.

#### Dopamine and its precursors

**Fig. 4:**
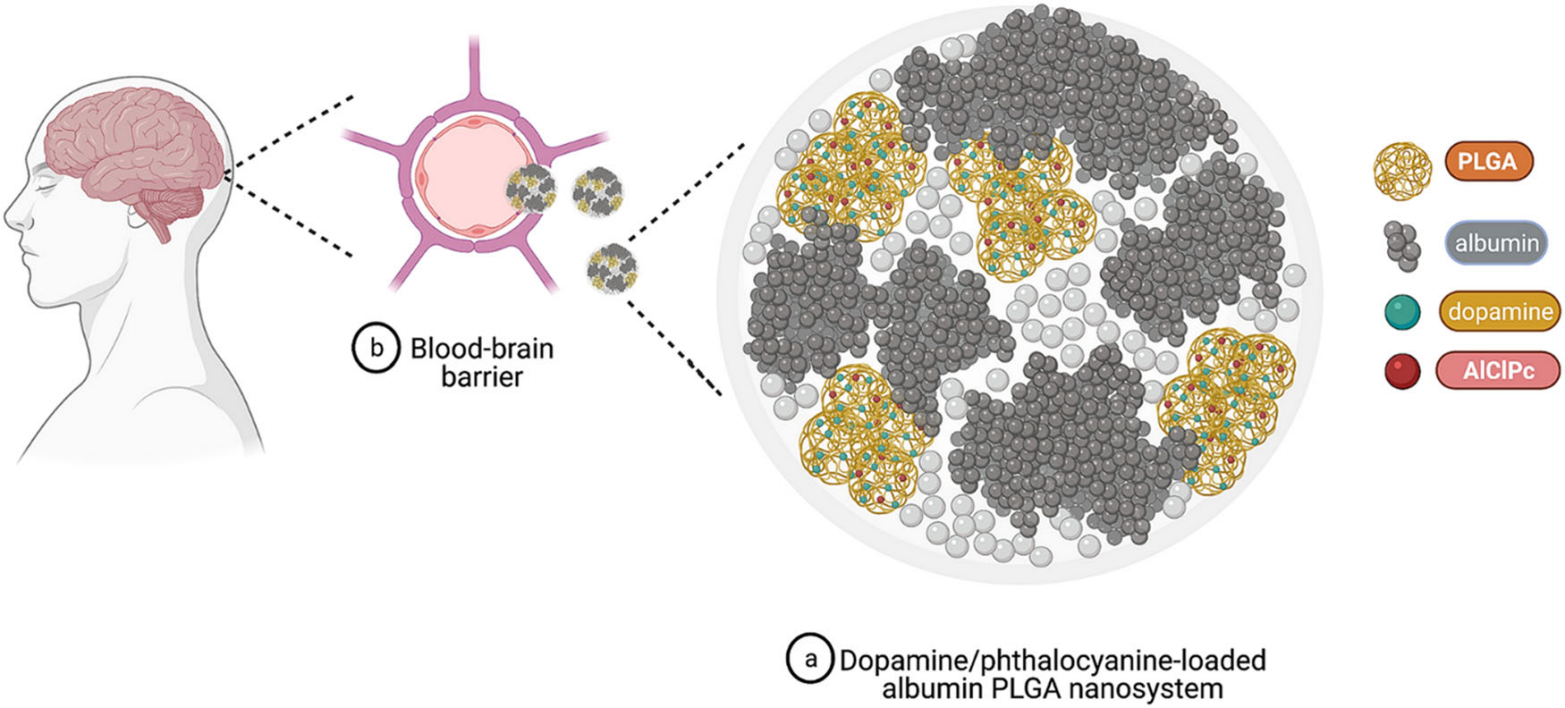
Dopamine/phthalocyanine-loaded albumin PLGA nanosystem designed to overcome the BBB for effective brain-targeted dopamine delivery. This innovative nanosystem combines biocompatible PLGA polymer, albumin for receptor-mediated BBB crossing, dopamine for neurotherapeutic efficacy, and aluminum chloride phthalocyanine (AlClPc) for fluorescent tracking. Reprinted with permission from ref. ^121^, Nature, 2021.

Monge-Fuentes et al. developed dopamine-loaded albumin/PLGA nanoparticles (DA-PLGA) to alleviate PD symptoms (Fig. 4)^121^. The nanoparticles were synthesized using a double emulsion-solvent evaporation method, then functionalized with albumin to enhance BBB permeability. The nanoparticles exhibited a diameter between 353 and 497 nm, with a PDI ranging from 0.4 to 0.6. Their zeta potential was negative (−27 to −37 mV), ensuring stability and favoring BBB crossing. The DA-PLGA were administered intraperitoneally at doses of 10 mg or 20 mg per animal in a 6-hydroxydopamine mouse model of PD. Behavioral and motor tests quantified their therapeutic efficacy. In the rotarod test, animals treated with 20 mg DA-PLGA showed a significantly prolonged latency to fall compared to untreated mice and levodopa-treated controls (*p* < 0.001). Similarly, in the adhesive removal test, DA-PLGA-treated mice removed adhesive from their forepaws in significantly less time (mean latency <50 s) compared to untreated lesioned mice (>100 s, *p* < 0.01), demonstrating restored sensorimotor function. In the apomorphine-induced rotation test, the number of rotations was significantly reduced in DA-PLGA-treated groups (~50% reduction, *p* < 0.001) compared to untreated lesioned animals, indicating improved motor asymmetry. Histological analysis using tyrosine hydroxylase immunohistochemistry confirmed the delivery and replenishment of dopamine in the nigrostriatal pathway but did not show neuroprotective effects against dopaminergic neuronal loss. Importantly, DA-PLGA successfully crossed the BBB, as confirmed by fluorescence imaging, and targeted delivery to the brain was achieved without systemic toxicity. These findings demonstrate the efficacy of DA-PLGA in mitigating PD symptoms while reducing systemic side effects^121^.

Another study by Danz et al. evaluated the development and characterization of dopamine-loaded PLGA (D-PLGA) nanoparticles for PD treatment^122^. The nanoparticles were synthesized using double-emulsion solvent evaporation method, resulting in nanoparticles with an average size of 198.1 ± 3.4 nm and PDI of 0.03 ± 0.01. Biological evaluations were conducted using both in vitro and advanced in vitro BBB models. Cytotoxicity tests on human brain microvascular endothelial cells (hBMECs) revealed no significant cell death, even at dopamine concentrations up to 25 µg/mL, demonstrating the biocompatibility of the formulation. Uptake studies showed a high level of nanoparticle internalization in immortalized mouse brain endothelial cell line (bEnd.3 cells), which represent the BBB (89.2% uptake), and substantial uptake in differentiated Lund human mesencephalic cells (LUHMES) around (64.9% uptake), indicating the targeting efficiency toward dopaminergic neurons. In the transport study using an advanced human induced pluripotent stem cells (hiPSC)-derived BBB model, 0.94% of the applied dopamine successfully crossed the barrier within 4 h. These findings support the safe and efficient dopamine delivery using PLGA nanoparticles for PD therapeutic applications^122^.

Parthipan et al. loaded levodopa-carbidopa in PLGA microparticles for oral administration, with the aim of achieving controlled and sustained drug release^123^. The resulting microparticles had an average size of 14 ± 1.8 μm, with a PDI of ~0.12, indicating a highly monodisperse population. In vitro tests were conducted to evaluate drug encapsulation efficiency and release kinetics. Drug encapsulation efficiency was >97% for levodopa and >68% for carbidopa. In vitro drug release studies in simulated intestinal fluid (SIF) and gastric fluids (SGF) revealed that over 80% of the two drugs were released within 5 h in SGF, with a sustained release observed up to 24 h in SIF. This release profile was consistent with a Ritger–Peppas model, suggesting that the transport mechanism involves diffusion, swelling, and polymer degradation. Compared to commercially available tablets, which release the two drugs within 2 h, this bicompartmental system provides a more controlled and sustained release. Such release behavior could reduce the frequency of dosing and the risk of side effects, such as dopamine-induced dyskinesia, while enhancing drug bioavailability^123^.

Another research group investigated the use of levodopa-benserazide loaded PLGA nanocarrier for preventing Levodopa-induced dyskinesia in PD-induced Sprague-Dawley rat models^124^. Benserazide is a peripheral dopa decarboxylase inhibitor that inhibits the conversion of levodopa to dopamine outside the brain. For this purpose, these particles were synthesized using solvent evaporation method. The resulting nanoparticles had an average size of ~500 nm, providing a controlled-release profile over 2 weeks without an initial burst effect. The prepared nanocarriers were administered subcutaneously at varying doses (20 mg/kg, 40 mg/kg, and 60 mg/kg), once a week for 3 weeks. In vitro drug release profiles demonstrated a steady release of the drugs over 2 weeks, and in vivo behavioral and molecular tests were conducted to assess efficacy and biological responses. This treatment significantly reduced levodopa-induced dyskinesia scores by up to 46% in peak abnormal involuntary movements scores and 60% in total abnormal involuntary movements scores at the optimal dose (40 mg/kg), compared to free levodopa administration. Additionally, molecular analysis indicated that up-regulation of β-arrestin2, an intracellular protein involved in the desensitization and internalization of dopamine receptors and activation of alternative signaling pathways, may be associated with reduced severity of dyskinesia in levodopa-benserazide-loaded PLGA nanocarrier-treated PD rats. This would suggest the role of β-arrestin2 in the stabilization of dopamine receptor signaling and overcoming the plasticity induced by chronic levodopa treatment. Genetic ablation of β-arrestin2 completely prevented the antidyskinetic effect of these nanocarriers, while its overexpression potentiated this therapeutic effect. These findings suggest that this PLGA-based nanosystem offers a novel and effective strategy for sustained PD therapy, improving patient outcomes by addressing both motor symptoms and therapy-induced side effects^124^.

#### Dopamine agonist

Li et al. developed a PLGA-based nanocarrier for PD therapy, combining pramipexole (PX, a dopamine agonist) with a photothermal agent (Au nanospheres) encapsulated in PLGA nanoparticles (Au-PX-PLGA)^125^. Solvent evaporation method was used for the nanoparticle preparation, producing uniform particles averaging 24 µm in size. Encapsulation efficiencies were 51.71 ± 0.54% for PX and 65.15 ± 2.30% for Au nanospheres. Upon near-infrared light irradiation, the Au nanospheres facilitated photothermal activation, enabling rapid and controllable drug release. For instance, cumulative drug release reached 24.7% after 8 min of irradiation at 2.5 W power. Biological testing involved both in vitro and in vivo evaluations. Cytotoxicity assays conducted on 3T3 fibroblasts, C2C12 myotubes, PC12 neurons, and RAW macrophages, demonstrated that neither Au nanospheres nor Au-PX-PLGA (0.01–0.5 mg/mL) caused significant toxicity, with cell viability remaining above 95%. In vivo pharmacokinetics, conducted in Sprague-Dawley rats, showed sustained PX plasma levels over two weeks, with a maximum plasma concentration of 19.06 ± 4.73 ng/mL observed at 48 ± 0.25 h*. Intramuscular* injection of Au-PX-PLGA nanoparticles in C57BL/6 mice assessed motor function and striatal dopamine restoration. The rotarod test demonstrated significant recovery in mice treated with Au-PX-PLGA, with fall latency increasing from 66.00 ± 37.18 s (untreated control group) to 291.17 ± 8.66 s after 14 days, indicating a recovery in motor abilities. Striatal dopamine levels reached 7.14 ± 1.19 ng/mg tissue, alongside significant increases in metabolites such as homovanillic acid (3.59 ± 0.51 ng/mg compared to 1.93 ± 0.43 ng/mg in controls) and 3,4-dihydroxyphenylacetic acid (13.43 ± 1.62 ng/mg compared to 8.97 ± 1.49 ng/mg in controls). Furthermore, the immunohistochemistry analysis evaluated the protective effects of the Au-PX-PLGA on dopaminergic neurons in the striatum and tyrosine hydroxylase, which is an essential enzyme in dopamine synthesis, served as the biomarker for dopaminergic neuron integrity. For this purpose, they stained brain slices using 3,3′-diaminobenzidine (DAB), and tyrosine hydroxylase analyzed using a light microscope. Optical density of tyrosine hydroxylase significantly decreased in control untreated group to 2.08 compared to ~4.76 in Au-PX-PLGA-treated group (*p* < 0.001). This was confirmed by the dark brownish-yellow staining in striatum section images, indicating high tyrosine hydroxylase levels. The study demonstrated that Au-PX-PLGA enhances dopamine restoration and motor recovery in PD models^125^.

**Fig. 5:**
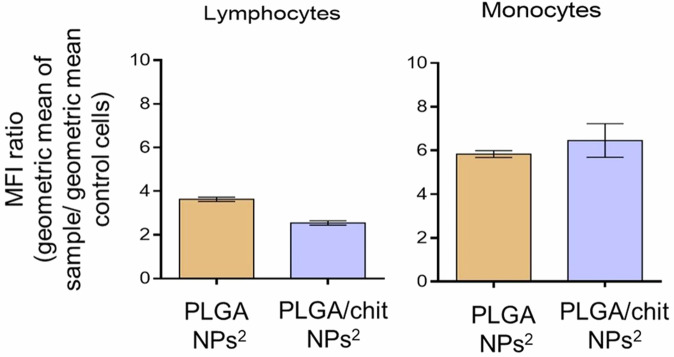
Enhanced uptake of Rhodamine B-loaded RH-PLGA and RH-PLGA/chitosan nanoparticles by lymphocytes and monocytes supporting their potential for targeted brain delivery. Quantitative analysis of mean fluorescence intensity demonstrates significant internalization of nanoparticles by lymphocytes and monocytes, with RH-PLGA/chitosan showing improved uptake in monocytes. Reprinted with permission from ref. ^126^, Elsevier, 2020.

Ropinirole (RH), another dopamine agonist, was encapsulated in chitosan coated PLGA nanoparticles to be delivered intranasally to the CNS for treatment of PD. This strategy aimed to overcome first-pass hepatic metabolism and limited BBB penetration^126^. The nanoparticles were fabricated using the nanoprecipitation technique, and the resulting RH-PLGA/chitosan nanoparticles had an average size of 468.0 nm and a PDI of 0.29. The controlled-release profile over 24 h showed up to 89% RH release from RH-PLGA/chitosan nanoparticles compared to only 20% RH release from PLGA nanoparticles with no chitosan. Cytotoxicity assays of RH-PLGA/chitosan nanoparticles using peripheral blood mononuclear cells and Raw 264.7 macrophages showed IC50 values of 1414 μg/mL and 19.3 μg/mL, respectively. ROS assay confirmed that the RH-PLGA/chitosan did not induce any oxidative stress. Sheep nasal mucosa was used for ex vivo permeability studies and revealed that RH-PLGA/chitosan increased RH permeability by 3.22-fold compared to RH-PLGA nanoparticles. Both RH-PLGA and RH-PLGA/chitosan caused minimal tissue disruption. The epithelial layer remained intact, with no significant changes in goblet cell distribution or mucus production. Additionally, cellular uptake studies confirmed enhanced internalization of RH-PLGA/chitosan into peripheral immune cells, as visualized by confocal microscopy and quantified by fluorescence intensity in lymphocytes and monocytes (Fig. 5). This enhanced uptake underscores the potential of surface modification of PLGA nanoparticles using chitosan to improve cellular interaction and CNS targeting via the intranasal route. This system holds promise for effective delivery of therapeutics to treat PD as well as other CNS disorders^126^.

**Fig. 6:**
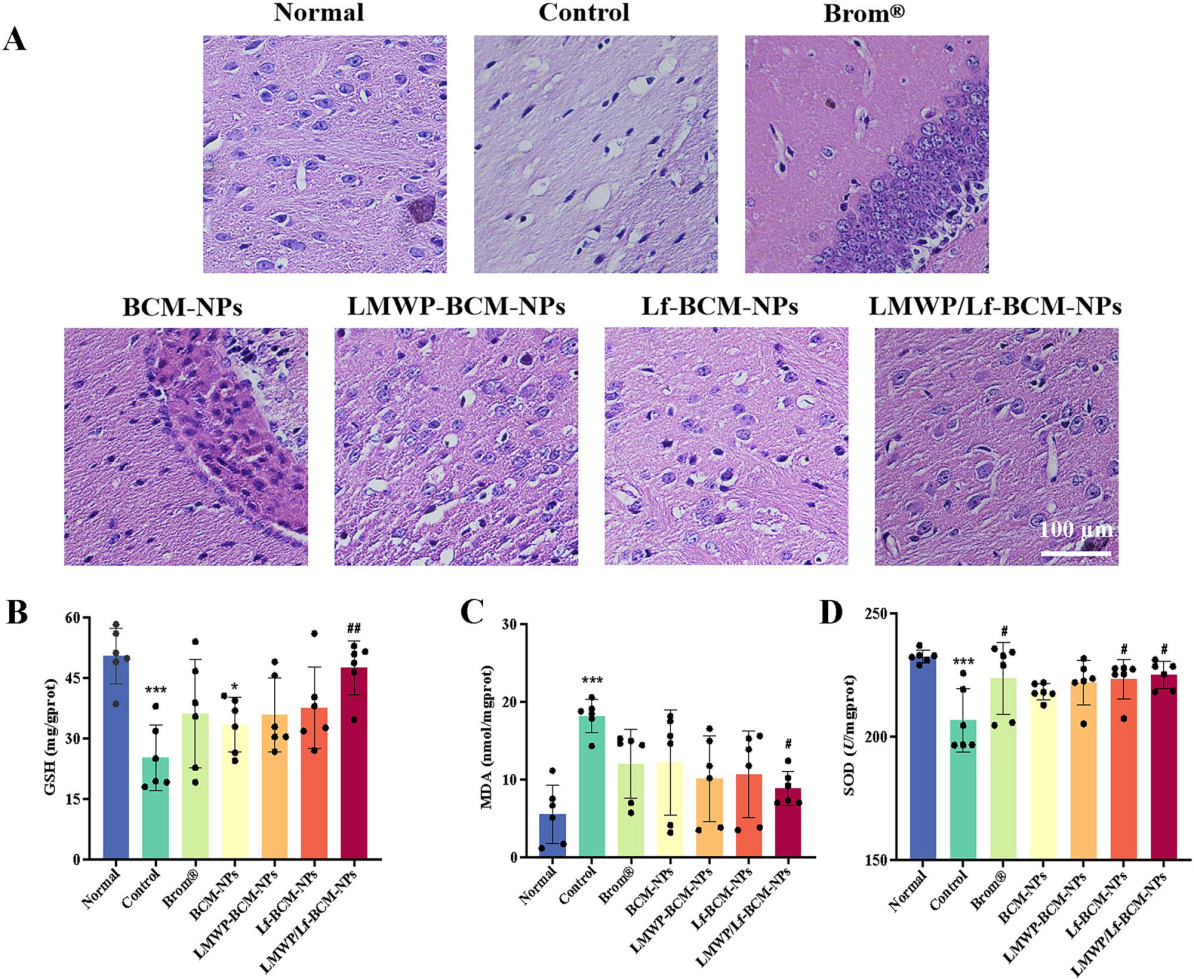
Histological evidence and biochemical markers showing neuroprotection and antioxidant activity in the brains of haloperidol-induced PD mice model following treatment with bromocriptine mesylate-loaded nanoparticle formulations. **A** Hematoxylin and Eosin-stained Cornu Ammonis 1 (hippocampal region) from different treatment groups. Normal group showed intact architecture of neurons. The control group (haloperidol-induced PD model, no treatment) displayed extensive neurodegeneration. The Brom® group (oral commercial bromocriptine mesylate, 2.5 mg/kg) shows partial neuronal restoration. The intranasal administration of bromocriptine mesylate-loaded PLGA nanoparticles (BCM-NPs) showed moderate protection. The nanoparticles modified with only low molecular weight protamine (LMWP-BCM-NPs) and modified with only lactoferrin (Lf-BCM-NPs) showed improved neuronal integrity. The dual-modified nanoparticles with both LMWP and Lf (LMWP/Lf-BCM-NPs) exhibited the most significant neuroprotection, with neuronal morphology comparable to the normal group. **B** Improved GSH levels, indicating enhanced antioxidant defense upon treatment with MWP/Lf-BCM-NPs (near-normal levels). **C** Significant reduction of MDA levels, showing reduced lipid peroxidation and oxidative stress with MWP/Lf-BCM-NPs. **D** Elevated SOD activity, supporting oxidative stress mitigation approaching the normal levels with MWP/Lf-BCM-NPs. Reproduced with permission from ref. ^127^, Elsevier, 2025.

Cong et al. encapsulated another dopamine agonist (Bromocriptine mesylate (BM)) into PLGA nanoparticles co-modified with lactoferrin and low molecular weight protamine (LMWP) (LMWP-lactoferrin-BM-loaded PLGA) for intranasal treatment of PD^127^. The PLGA nanoparticles were fabricated using single emulsion solvent evaporation method, then LMWP and lactoferrin were conjugated onto the surface of PLGA nanoparticles. TEM imaging confirmed the uniform morphology of the nanoparticles that are spherical, while DLS analysis showed a particle size of 248.53 ± 16.25 nm with a PDI of 0.274 and a zeta potential of −2.63 ± 0.74 mV, confirming the colloidal stability. High EE% was obtained (73.30 ± 0.74%) with DL% of 10.11 ± 0.10%. FTIR analysis confirmed the successful conjugation of LMWP and lactoferrin. A Transwell system (mimics the physiological barrier between two cellular compartments) was utilized to evaluate the permeability across BBB in an in vitro model utilizing brain capillary endothelial cells (BCECs). The co-modified nanoparticles exhibited 6.53-fold higher fluorescence intensity in BCECs compared to unmodified BM-loaded PLGA nanoparticles. MTT assay showed more than 70% cell viability on human bronchial epithelial cell line (16HBE14o-) and BCECs, indicating biosafety. Haloperidol-induced PD mice models (Swiss albino mice) were utilized for the in vivo assessment through intranasal administration of the prepared nanoparticles. Behavioral assessment of control vs. LMWP-lactoferrin-BM-loaded PLGA included akinesia test (reduced from 86.74 s vs. 21.88 s), catalepsy test (decreased from 102.87 s vs. 21.88 ± 2.25 s), forced swim test (swimming time improved from 63.05 s vs. 108.39 s), and grip strength test (enhanced from 0.5 vs. 3.67), respectively. Preservation of neuronal morphology was observed in Cornu Ammonis 1 (hippocampal region) in co-modified-BM-PLGA-treated group compared to control through histopathological analysis. Enhanced antioxidant defense was confirmed by biochemical analysis (Fig. 6) where glutathione (GSH) levels were elevated by 2-fold (47.61 ± 6.73 mg/gprot) compared to control and superoxide dismutase (SOD) activity increased to 225.12 ± 5.50 U/mgprot (vs 206.68 ± 12.90), while malondialdehyde (MDA) levels were reduced to 8.88 ± 2.19 nmol/mgprot (vs 18.21 ± 2.10 in control), confirming the reduction of lipid peroxidation. Maximum brain accumulation was observed at 2 h post administration through fluorescence imaging, suggesting enhanced nose to brain targeting efficiency. Moreover, no signs of inflammation were observed in nasal mucosa of co-modified-BM-loaded PLGA-treated mice, suggesting a safe and non-invasive strategy for intranasal delivery^127^.

#### MAO-B inhibitors

**Fig. 7:**
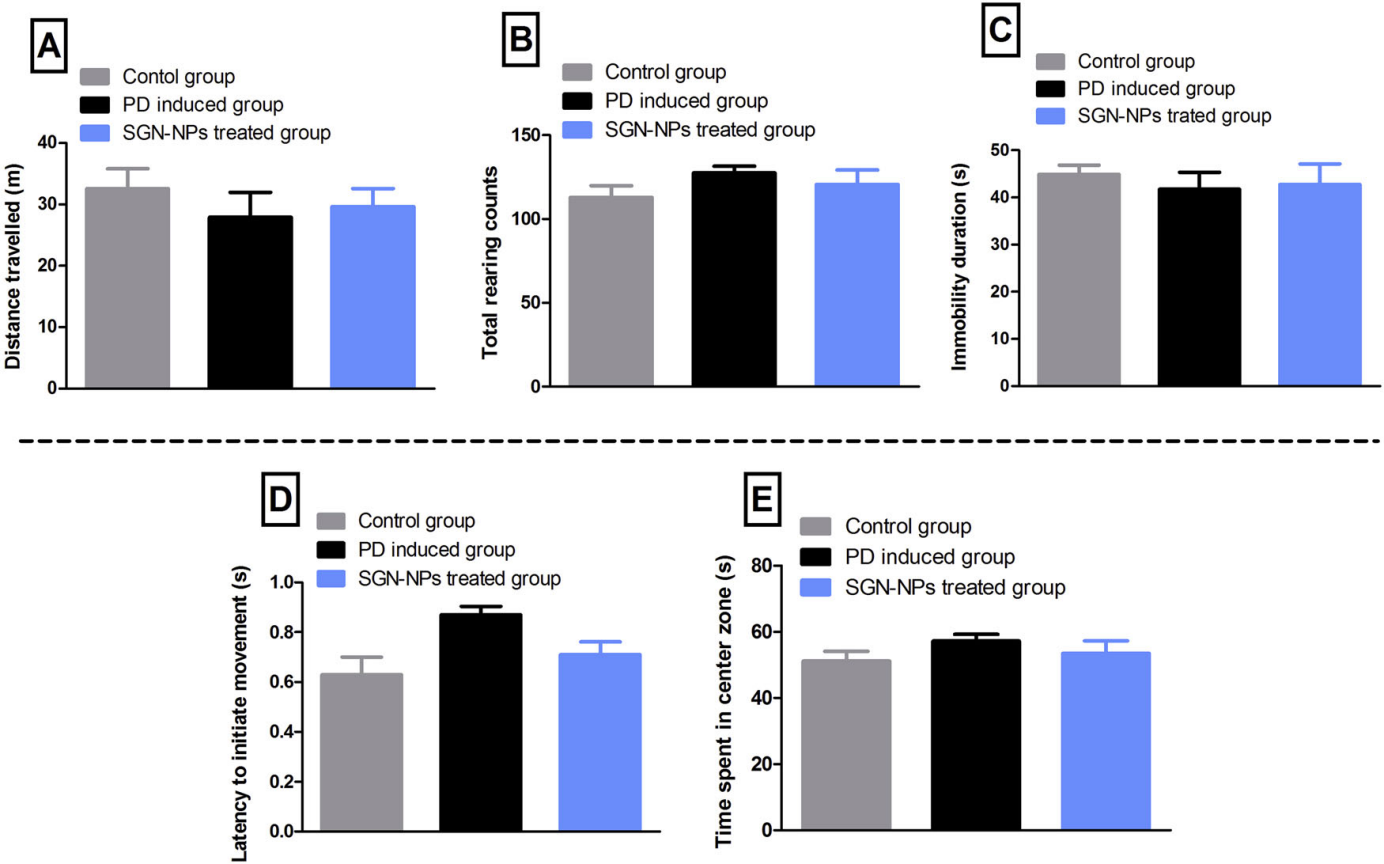
Behavioral performance assessment in control, PD-induced, and rats treated with PLGA loaded with selegiline. **A** Distance traveled, **B** rearing counts, **C** immobility duration, **D** latency to initiate movement, and **E** time spent in the center zone were recorded. The PD-induced group exhibited motor deficits compared to the control group, validating disease induction. Treatment with S-PLGA transdermal films reversed these impairments, as evidenced by improved movement initiation and normalized behavior. S-PLGA were shown to restore the functional motor movements supporting their potential for PD. Reprinted with permission from ref. ^128^, Elsevier, 2019.

Balie and Salve investigated the development of transdermal films embedded with selegiline (a selective inhibitor of MAO-B taken in combination with levodopa to slow progression of PD) loaded into PLGA nanoparticles (S-PLGA) for sustained treatment of PD^128^. The nanoparticles, synthesized using a double emulsion solvent evaporation technique, demonstrated a mean particle size of 73.8 nm, a PDI of 0.461, and a zeta potential of −29.11 mV. After incorporation into ethylene-vinyl acetate transdermal films via solvent casting, their characteristics were retained, with uniform drug distribution across films. Pharmacokinetic studies in Wistar rats revealed an enhancement in drug absorption and prolonged systemic presence after transdermal application. For selegiline, the area under the curve upon using S-PLGA transdermal films increased 13-fold compared to intravenous administration. In vivo biodistribution studies showed effective brain targeting, with S-PLGA transdermal films achieving higher brain drug concentrations over 36 h. Biological efficacy was evaluated using a reserpine-induced PD rat model. The application of S-PLGA transdermal films reduced MAO-B levels in brain tissues from 215.11 ± 23.19 ng/g in untreated PD rats to 114.75 ± 11.63 ng/g. Simultaneously, dopamine levels increased significantly from 29.17 ± 6.39 ng/g in PD rats to 102.31 ± 7.51 ng/g after treatment, though they remained below the control group level (135.90 ± 9.21 ng/g). Behavioral assessments revealed improvements in cataleptic activity, with reduced immobility time observed following treatment, as shown in Fig. 7. Additionally, skin irritation tests demonstrated the transdermal films were well-tolerated, showing no signs of erythema or edema even after 72 h of application. These results suggest that S-PLGA transdermal films provide a promising, non-invasive, and sustained delivery system for treating PD^128^.

#### COMT inhibitors

**Fig. 8:**
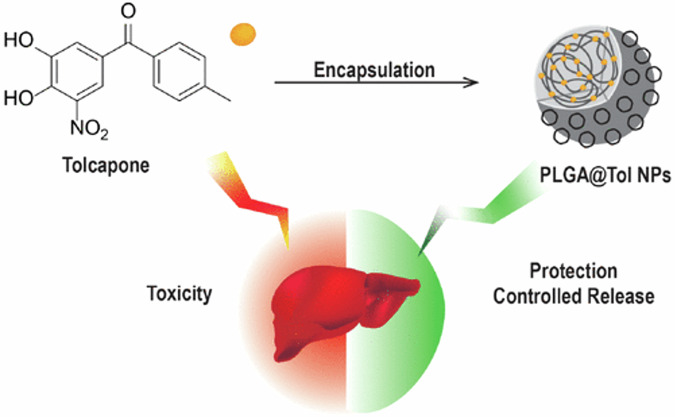
Encapsulation of tolcapone into PEGylated PLGA nanoparticles (PLGA@Tol NPs), demonstrating the dual benefits of mitigating hepatotoxicity in HepG2 cells while enabling controlled drug release for safer and possible therapeutic application in PD. Reprinted with permission from ref. ^129^, American Chemical Society, 2024.

Two studies evaluated the encapsulation of entacapone and tolcapone in PEGylated PLGA nanoparticles. First, Pinto et al. developed tolcapone-loaded PLGA nanosystem to reduce the hepatotoxic effect of entacapone while retaining its therapeutic efficacy in PD as illustrated in Fig. 8^129^. The nanoparticles were prepared using the nanoprecipitation method, achieving an average particle size of 148 nm, with a PDI of <0.2. Biological evaluations were conducted using human hepatocarcinoma cell line (HepG2 cells) under two conditions: glucose-containing and glucose-free (galactose-supplemented) media to model mitochondrial toxicity. Cytotoxicity assays, including MTT and SRB, showed significantly reduced toxicity for the PEGylated nanoparticle formulation compared to free tolcapone (cell viability exceeded 85% at 10, 25, and 50 µM of the formula after 24 and 48 h of treatment). Additionally, ATP depletion was effectively mitigated, and ROS production was reduced, with the nanoparticle system demonstrating lower ROS levels compared to untreated cells. In vitro COMT inhibition assays demonstrated that the tolcapone-loaded PLGA nanoparticles achieved comparable enzyme inhibition efficacy to free tolcapone, confirming therapeutic retention which was achieved in addition to a controlled-release profile and reduced cytotoxicity. The study highlights that PEGylated PLGA nanoparticles, could offer a promising alternative for the safe delivery of tolcapone, maintaining its therapeutic effects while significantly reducing associated hepatotoxicity^129^.

**Fig. 9:**
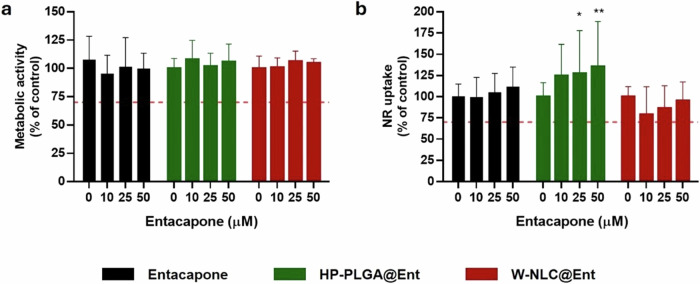
Assessment of viability and lysosomal activity in SH-SY5Y cells treated with free entacapone, NLC@Ent, and HP-PLGA@Ent. Metabolic and lysosomal activities were evaluated using Alamar-Blue reduction assay (a) and neutral red uptake (NR uptake) (b) assay, respectively, in differentiated SH-SY5Y cells treated with free entacapone, PLGA@Ent, and NLC@Ent nanoparticles. All formulations demonstrated cell viability above 85%, with a significant increase in lysosomal activity observed for PLGA@Ent at higher entacapone concentrations (p < 0.01). HP-PLGA@Ent: PLGA nanoparticles loaded with entacapone and lyophilized with the addition of 2-hydroxypropyl-β-cyclodextrin as a cryoprotectant. Reprinted with permission from ref. ^130^, ACS, 2024.

Second, Machado et al. encapsulated entacapone into PEGylated PLGA (PEG-PLGA@Ent) nanoparticles and nanostructured lipid carriers (NLCs) to enhance its bioavailability and pharmacological efficacy while mitigating its limitations, such as low oral bioavailability and rapid clearance^130^. PEG-PLGA@Ent nanoparticles were prepared using the nanoprecipitation method, yielding a size of 186.0 ± 4.0 nm. The in vitro release profile showed an initial burst of 29.3 ± 4.0% of entacapone released from PEG-PLGA@Ent in the first 2 h, with a cumulative release of 39.7% over 72 h. Biological evaluation included in vitro cytotoxicity tests on HepG2, intestinal epithelial cell line (Caco-2), and dopaminergic neurons (Differentiated SH-SY5Y with 0.1% retinoic acid) cell lines, showing low cytotoxicity. As depicted in Fig. 9, metabolic activity tested using Alamar-Blue reduction assay in SH-SY5Y cells remained above 85% for all entacapone concentrations tested. Additionally, lysosomal activity (neutral red uptake) was slightly enhanced at higher concentrations with PLGA@Ent formulations (*p* < 0.01), further supporting the biocompatibility of these nanoparticles. The COMT inhibition assay in HepG2 cells using the 3-BTD fluorescent probe showed inhibition levels of 78.5–79.6%, comparable to free entacapone, confirming the preservation of its therapeutic efficacy post-encapsulation. Overall, these findings underscore the potential of PLGA-based nanosystem to enhance the clinical utility of entacapone^130^.

#### HDAC6 inhibitors

**Fig. 10:**
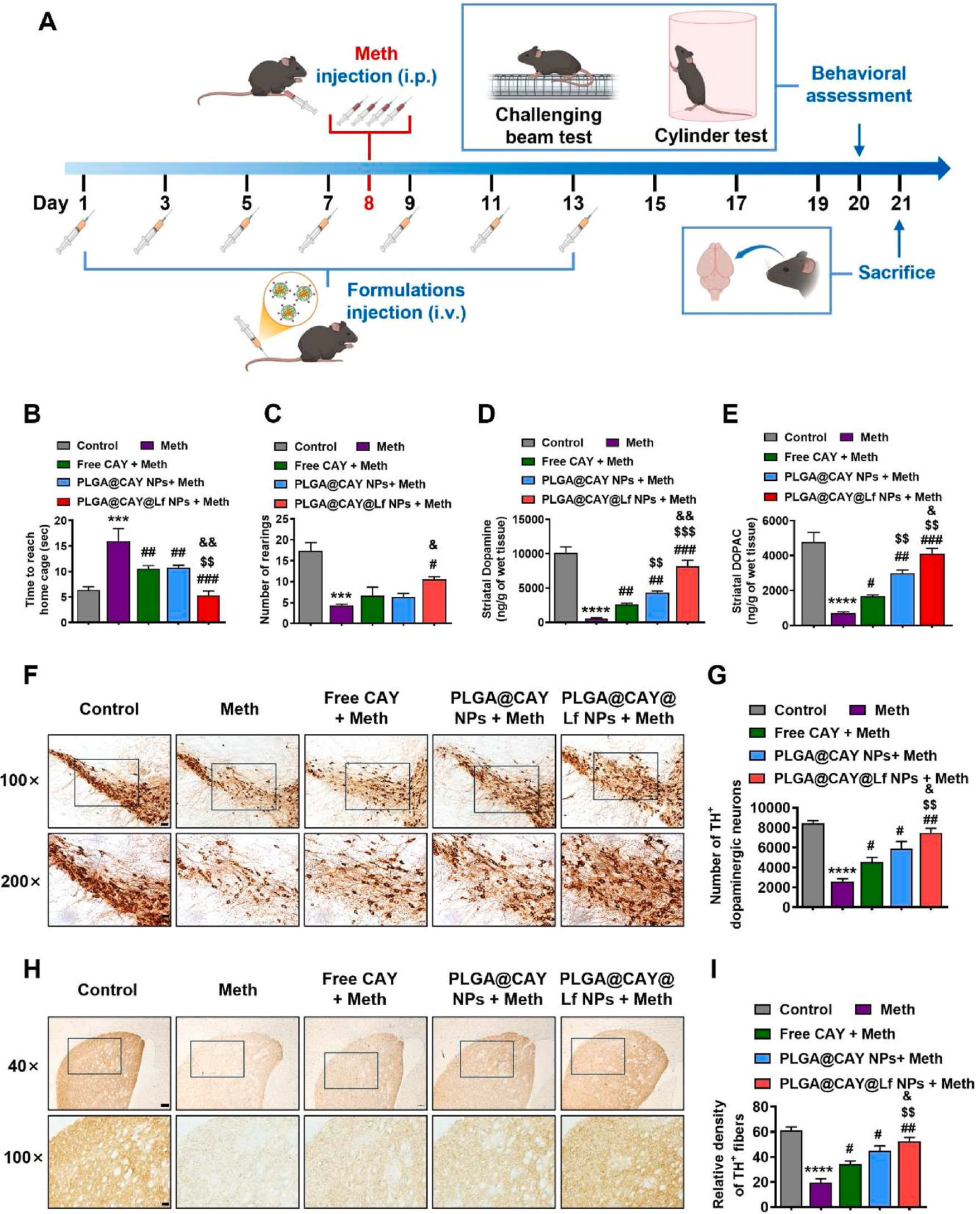
Therapeutic efficacy of lactoferrin-CAY10603-loaded PLGA nanoparticles in methamphetamine-induced PD mouse model. **A** Intravenous injections of lactofferin-CAY10603-loaded PLGA for 21 days and intraperitoneal methamphetamine injection on day 8. **B** Challenging beam test results showing time to reach the home cage, where methamphetamine significantly impaired motor coordination and lactofferin-CAY10603-loaded PLGA improved performance. **C** Cylinder test showing number of rearings (standing on the hind limbs as an indicator of motor activity) where methamphetamine-treated mice exhibited reduction in rearing frequency reflecting impaired motor function, whereas lactoferrin-CAY10603-loaded PLGA-treated mice significantly restored the rearing behavior indicating improved motor performance. **D**, **E** Restoration of striatal dopamine and 4-dihydroxyphenylacetic acid (DOPAC) levels in lactoferrin-CAY10603-loaded PLGA -treated mice compared to severe depletion in methamphetamine-treated group. **F**, **H** Tyrosine hydroxylase staining of substantia nigra and striatal regions, respectively, showing protection of dopaminergic neurons. **G**, **I** Quantification of tyrosine hydroxylase+ in dopaminergic neurons and assessment of fiber density confirming neuroprotection by lactofferin-CAY10603-loaded PLGA. Reproduced with permission from ref. ^20^, Elsevier, 2025.

To reduce rapid elimination and low brain concentration of CAY10603, Pham et al. encapsulated CAY10603 (selective HDAC6 inhibitor) in PLGA nanoparticles functionalized with lactoferrin to enhance BBB permeability (lactoferrin- CAY10603-PLGA)^20^. CAY10603-PLGA were fabricated using single-emulsion solvent evaporation method and then surface factionalized with lactoferrin. TEM imaging confirmed the spherical shape of the nanoparticles exhibiting a diameter of 99.7 ± 9.4 nm. DLS analysis indicated a PDI of 0.09 with Zeta potential value of −49.2 ± 2.9 mV, confirming high stability. FTIR spectroscopy validated CAY10603 loading and lactoferrin conjugation, while the EE% was determined to be 20.57 ± 1.21% with DL% of 0.69 ± 0.06%. Drug release profile showed an initial burst release of 25.1 ± 1.4%, followed by sustained release over 192 h. Cellular uptake was evaluated in SH-SY5Y neuroblastoma and hBMECs using confocal laser scanning microscopy and flow cytometry revealing 5.4X higher uptake of lactoferrin-conjugated nanoparticles as compared to non-conjugated PLGA NPs following 5 h of exposure. Furthermore, pretreatment with free lactoferrin reduced this uptake, confirming lactoferrin receptor-mediated endocytosis. Trans-endothelial electrical resistance (TEER) was evaluated on hBMEC monolayers showing a value of 149.7 ± 3.6 Ω·cm² by day 6, indicating the formation of a tight barrier mimicking the BBB in an in vitro model, thereby confirming the validity of subsequent permeability study. The permeability study demonstrated enhanced BBB (in vitro model) permeability mediated by lactoferrin receptor-targeted transcytosis, showing a 2.7-fold increase in fluorescence intensity for lactoferrin-conjugated nanoparticles compared to non-functionalized ones. Cell Counting Kit-8 (CCK-8) was utilized on methamphetamine-treated SH-SY5Y cells (to mimic methamphetamine-induced PD model), where lactoferrin- CAY10603-PLGA showed 86.3% cell viability. Live/dead staining further confirmed reduced apoptosis. Mitochondrial membrane potential was evaluated through JC-1 assay on methamphetamine-treated SH-SY5Y cells, where pretreatment with lactoferrin- CAY10603-PLGA showed restoration of mitochondrial membrane potential, demonstrating effective reversal of mitochondrial dysfunction. Intracellular ROS levels were assessed using 2′,7′-dichlorodihydrofluorescein diacetate (DCFH-DA) staining and flow cytometry, revealing a 9.1-fold increase in ROS production in methamphetamine-treated SH-SY5Y cells. Pretreatment with lactoferrin-CAY10603-loaded PLGA nanoparticles significantly reduced ROS levels by ~3.0-fold, indicating strong antioxidant activity. Immunofluorescence and Western blotting in SH-SY5Y cells revealed a 4.5-fold reduction in acetylated α-tubulin following methamphetamine treatment, which was reversed by11.9-fold upon lactoferrin-CAY10603-PLGA treatment. Similarly, α-synuclein aggregation was reduced to 3.3-fold with lactoferrin- CAY10603-PLGA treatment. The prepared nanoparticles were tested using methamphetamine-induced PD in C57BL/6J female mice^20^. Lactoferrin-conjugated nanoparticles treatment were introduced via intravenous injection and showed higher brain accumulation than unmodified nanoparticles by 1.5 folds (Fig. 10). Behavioral assessments showed that lactoferrin- CAY10603-PLGA improved locomotor performance in the challenging beam and cylinder tests, while dopamine levels were increased to 8194 ± 840 ng/g, compared to 591 ± 103 ng/g in control mice. Tyrosine hydroxylase immunostaining revealed a significant preservation of dopaminergic neurons, with density recovery to 52.5 ± 3.0 in striatum (vs. 19.4 ± 3.2 in methamphetamine-treated mice). α-synuclein levels decreased by 17.9-fold and acetyl-α-tubulin increased by 4.2-fold in lactoferrin- CAY10603-PLGA-treatd group, compared to untreated group. Immunostaining for glial fibrillary acidic protein (GFAB, a marker for astrocytes) and ionized calcium-binding adapter molecule 1 (Iba-1, a marker for microglia) indicated reduced astrocyte and microglia activation in both striatum and substantia nigra. Hemolysis rate was <5%, while Hematoxylin and Eosin staining revealed no histopathological abnormalities in different organs, confirming biosafety. These findings demonstrate that lactoferrin-PLGA nanoparticles enhanced the brain delivery of CAY10603, improving mitochondrial function and limiting the oxidative stress and α-synuclein aggregation^20^.

#### Biguanide-derived neuroprotective agent (metformin)

To mitigate neuroinflammation and mitochondrial dysfunction in PD, metformin was encapsulated into PLGA, which were further coated with BV2 microglial cell membranes to enhance the targeting efficiency to neuroinflammatory sites (BV2-metformin-loaded PLGA) for intranasal administration^19^. The nanoparticles were synthesized using double emulsion solvent evaporation method. Then, BV2 microglial membranes were coated on their surface using ultrasonication and extrusion. TEM imaging confirmed the core-shell structure, while DLS analysis revealed a particle size of 252.2 ± 7.1 nm with PDI of 0.228 ± 0.011 and zeta potential of −20.6 ± 1.1 mV. EE% and DL% were 10.76 ± 0.72% and 3.98 ± 0.34%, respectively. Sodium dodecyl sulfate–polyacrylamide gel electrophoresis (SDS-PAGE) verified the characteristic protein bands of BV2 microglial membranes. FTIR analysis confirmed distinct peaks of the formula components. In vitro CCK-8 assay of SH-SY5Y (neuronal) and BV2 (microglial) cells demonstrated low cytotoxicity up to 0.5 mM. To induce oxidative stress, both cell lines were treated with 1-methyl-4-phenylpyridinium (MPP^+^, a neurotoxic compound that causes mitochondrial dysfunction). ROS levels in these cells were assessed using DCFH-DA staining followed by fluorescence microscopy, revealing that BV2-metformin-loaded PLGA reduced intracellular ROS compared to uncoated nanoparticles and restored mitochondrial membrane potential (MMP) via JC-1 staining. Autophagy was enhanced in MPP+-treated SH-SY5Y cells upon treatment with coated nanoparticles, as confirmed by monodansylcadaverine (MDC) staining. Higher uptake of BV2-metformin-loaded PLGA was observed in BV2 cells compared to SH-SY5Y, indicating preferential targeting to microglia. In BV2 microglial cells, BV2-metformin-loaded PLGA treatment downregulated inducible nitric oxide synthase (iNOS) expression (proinflammatory M1 phenotype) and upregulated mannose receptor (CD206) expression (anti-inflammatory M2 phenotype), leading to a more than three-fold elevation in the CD206/iNOS fluorescence intensity ratio. For the in vivo study, PD was induced in male C57BL/6 mice by 1-methyl-4-phenyl-1,2,3,6-tetrahydropyridine (MPTP). PD-induced mice model was treated with BV2-metformin-loaded PLGA 3 times per week for 3–4 weeks. Brain-targeting efficiency was assessed and showed a peak of BV2-metformin-loaded PLGA at 12 h which was sustained up to 48 h post-administration. For behavioral assessment, rotarod test increased from 2 s (pre-treatment) to 60 s (post-treatment), approaching normal performance. The descent time in pole test was shortened in BV2-metformin-loaded PLGA-treated mice compared to untreated PD-mice, and suspension test improved grip score (from 1 to 3 points). Immunohistochemistry revealed that tyrosine hydroxylase positive neurons in the substantia nigra were significantly restored. ELISA assays showed that BV2-metformin-loaded PLGA lowered pro-inflammatory markers (TNF-α, IL-6) and boosted anti-inflammatory markers (IL-4, IL-10) in both the brain tissue and blood samples^19^. To understand the molecular mechanisms of BV2-metformin-loaded PLGA on midbrain tissues, transcriptomic analysis using RNA sequencing was conducted. Total RNA was extracted generating expression data for 32,598 genes^19^. Then, the differentially expressed genes (DEGs) were identified in treated and untreated PD-mice models. Results showed that 44 genes were upregulated and 43 genes were downregulated in the BV2-metformin-loaded PLGA-treated group, indicating that the treatment had a profound impact on gene expression. To explore the functional relevance of these DEGs, gene ontology (GO) enrichment analysis was conducted, revealing that the altered genes were primarily involved in immune response regulation, cytokine production, oxidative stress response, and mitochondrial functions. Moreover, Kyoto encyclopedia of genes and genomes (KEGG) pathway analysis was conducted for identification of the involved inflammatory signaling pathways. Findings of this analysis revealed the involvement of the NF-κB pathway, TNF signaling, and IL-17 pathway, suggesting that coated-PLGA modulate neuroinflammation and mitochondrial homeostasis. Immunofluorescence staining of brain tissue examined the expression of tumor necrotic factor-alpha (TNF-α, pro-inflammatory cytokine), glial fibrillary acidic protein (GFAP, a marker of activated astrocytes), sequestosome 1 (p62, marker of autophagic activity), voltage-dependent anion channel 1 (VDAC1, marker of mitochondrial stress), and NADPH Oxidase 4 (NOX4, marker of mitochondrial stress). The results demonstrated that BV2-metformin-loaded PLGA treatment reduced TNF-α and GFAP expression, confirming suppression of neuroinflammation, while p62 levels were increased, indicating enhanced autophagy. Mitochondrial stress markers VDAC1 and NADPH oxidase 4 were downregulated. Hematoxylin and eosin staining of major organs showed no observable toxicity, confirming systemic safety^19^.

### Natural products loaded PLGA nanoparticles for PD treatment

Research studies demonstrated the effectiveness of PLGA systems in encapsulating natural bioactive compounds, thereby improving bioavailability and BBB penetration. Table 2 provides a summary of the natural compounds, delivery systems, characterization techniques, models, and key outcomes reported in PD studies.

**Table 2:** Encapsulation of natural compounds in PLGA-based nanosystems as proposed therapeutics for PD

Classification/compound name	Delivery system	Characteristics of the prepared nanoparticles	Cell line/animal model (route of administration)	Key outcomes	Ref
Polyphenol/Resveratrol	Lactoferrin-Resveratrol-PLGA	Particle size: 148.2 ± 4.2 nm. PDI: 0.12 ± 0.18. Zetapotential: −23.1 ± 3.0 mV. EE: 75.2 ± 4.1%. DL: 6.1 ± 0.3%. Release profile (72 h): 38.6 ± 2.5%. FTIR, DSC.	SH-SY5Y neuroblastoma cells, HBMECs (in vitro); C57BL/6 mice (intravenous)	-Enhanced BBB penetration -Reduced ROS and improved MMP -Dopamine restoration in striatum -Improved motor function in PD model -Reduced neuroinflammation and cytokine levels	^131^
Polyphenols/Curcumin	Curcumin-PLGA	Particle size: 436 ± 58.3 nm. PDI: 0.31 ± 0.16. Zetapotential: −27.5 ± 0.6 mV. EE: 88.23%. DL: 1.76%. Release profile (6 h): 30%.	C57BL/6 mice (intravenous injection via tail vein)	-Behavioral improvement in PD mouse model (rotarod test and climbing test) -Reduced neurodegeneration and improved drug delivery to deep brain regions	^132^
Flavonoids (flavanone)/Naringenin	Naringenin-PLGA	Particle size: 162.1 nm. PDI: 0.288. Zetapotential: −9.8 mV. EE: 91.21%. DL: 91%. Release profile (10 h): 54.62 ± 1.4%. XRD, DSC.	Wistar rats (oral administration)	-Enhanced antioxidant activity in brain tissue -Reduced MDA levels (lipid peroxidation) and increased SOD, CAT, and GSH activities. -Behavioral improvements in rotarod and open field tests. -Reduced α-synuclein levels and increased BDNF expression. -Histopathology showed protection against neuronal degeneration.	^133^
Flavonoid (isoflavone)/Puerarin	Puerarin-PLGA	Particle size: 88.36 ± 1.67 nm. PDI: 0.047 ± 0.007. Zetapotential: −18.85 ± 2.76 mV. EE: 89.52 ± 1.74%. DL: 42.97 ± 1.58%. Release profile (48 h): 93% drug release in SGF, and 96% in SIF. XRD, DSC.	MDCK cells (intestinal permeability studies), SH-SY5Y cell, Sprague-Dawley rats (oral), C57BL/6 mice (oral), Zebrafish embryos (toxicity and biodistribution)	- Increase in plasma bioavailability and brain accumulation compared to free form - Cell viability in MPP+ model - Reduction in tyrosine hydroxylase neuron loss in MPTP model - No significant toxicity in zebrafish.	^134^
Coumarins (benzopyrone)/coumarin	Coumarin-PEGylated-PLGA	Particle size: 104.6 ± 1.0 nm in PBS. PDI: 0.05 ± 0.01. Zetapotential: −10 mV. EE: 53.3 ± 2.0%. Release profile (24 h): 30%. ^1^HNMR, DSC.	SH-SY5Y, Caco-2, hCMEC/D3 cells, Rats (in vivo)	-Overcame P-gp-mediated efflux and enhanced cellular uptake. -Enhanced permeability across intestinal and brain endothelial barriers -Reduced cytotoxicity of free coumarin and maintained cell viability - Prolonged systemic circulation and increased bioavailability compared to free coumarin.	^135^
Flavonoids (flavan-3-ol)/Epigallocatechin gallate	Epigallocatechin gallate-PLGA	Particle size: 129.4 ± 15.5 nm. PDI: 0.077. Zetapotential: −18.1 ± 2.8 mV. EE: 86.27 ± 2.42%. DL: 13.02 ± 1.60%. Release profile (120 h): 49%. FTIR, TGA.	Nerve-like cells (exposed to rotenone)	Protected nerve-like cells from oxidative stress, prevented ROS, mitochondrial damage, and DNA fragmentation.	^161^
Flavonoids (flavonoid glycoside)/Rutin	Rutin-PLGA	–	6-OHDA-induced PD mouse model (intracerebroventricular injection)	-Improved behavioral parameters -Restored antioxidant enzyme levels (SOD, CAT, GPx), GSH, and dopamine/metabolites in the striatum while reducing ROS levels.	^162^
Flavonoid (dihydroflavonol)/Dihydroquercetin	Mannitol-Dihydroquercetin-Platinum based nanozymes-PLGA	Particle size: 210.6–236.1 nm. PDI: 0.077. Zetapotential: −26.19 ± 0.3 mV.	Microglia and neuronal cell lines; mouse model of PD (intravenous injection)	Synergistic antioxidative activity of Dihydroquercetin and Ptzymes. -Reduced oxidative stress-induced neuronal damage -Promoted M2-phenotype polarization of microglia -Suppressed neuroinflammation in brain	^163^
Terpenoids/Geraniol	Geraniol- Ursodeoxycholic acid-PLGA	Particle size: 181 ± 5.9 nm. PDI: 0.060 ± 0.02. Zetapotential: −26.7 ± 6.5 mV. EE: 89.3 ± 3.2%. DL: 12.1 ± 1.4%. Release profile (8 h): 40%.	Sprague-Dawley rats (nasal administration)	- Effective brain delivery, with the drug detected in cerebrospinal fluid minutes post-administration. -No significant damage to the nasal mucosa was observed.	^164^
Alkaloids/Nicotine	Nicotine-PLGA	–	In vitro release simulated cerebrospinal fluid	Zero-order nicotine release over 50 days -Minimal matrix degradation -Robust mechanical properties.	^165^

*6-OHDA* 6-Hydroxydopamine, *BBB* blood-brain barrier, *BDNF* brain-derived neurotrophic factor, *Caco-2* human colorectal adenocarcinoma cells, *CAT* catalase, *DL* drug loading capacity, *DSC* differential scanning calorimetry, *EE* encapsulation efficiency, *FTIR* Fourier transform infrared spectroscopy, *GSH* glutathione, *GPx* glutathione peroxidase, *hCMEC/D3* human cerebral microvascular endothelial cells, *HBMECs* human brain microvascular endothelial cells, *MDA* Malondialdehyde, *MDCK* Madin-Darby Canine kidney cells, *MMP* mitochondrial membrane potential, *MPTP* 1-Methyl-4-phenyl-1,2,3,6-tetrahydropyridine, *PDI* polydispersity index, *P-gp* P-glycoprotein, *Ptzymes* Platinum-based nanozymes, *ROS* reactive oxygen species, *SOD* superoxide dismutase, *TGA* thermogravimetric analysis, *XRD* X-ray diffraction.

#### Polyphenols

**Fig. 11:**
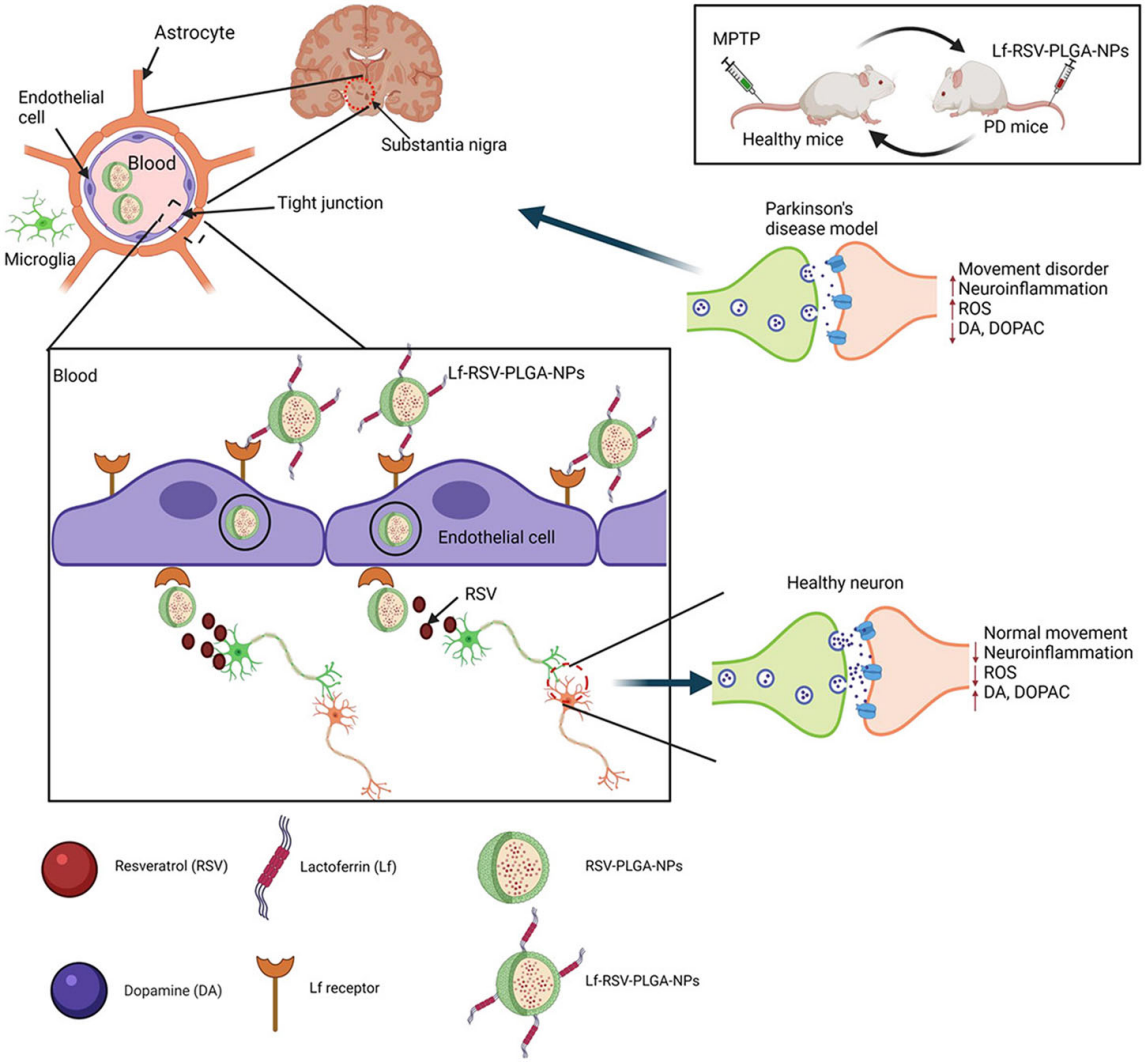
PLGA functionalized with lactoferrin and loaded with resveratrol for targeted neuroprotection in PD. The nanoparticles could cross the BBB via lactoferrin receptor-mediated transcytosis, delivering resveratrol directly to the substantia nigra. The prepared nanoparticles reduced ROS, neuroinflammation, and restored dopaminergic signaling in PD-induced mice, resulting in improved motor function. Reprinted with permission from ref. ^131^, Elsevier, 2022.

Katila et al. designed resveratrol-loaded PLGA nanoparticles functionalized with lactoferrin (Lf-RSV-PLGA-NPs), to enhance the neuroprotective effects against PD (Fig. 11)^131^. The nanoparticles were prepared using an emulsion solvent evaporation method, yielding particles with a mean size of 148.2 ± 4.2 nm, a PDI of 0.12, and a zeta potential of −23.1 ± 3.0 mV, supporting their suitability for BBB penetration. Sustained release kinetics were observed, with 38.6 ± 2.5% of encapsulated resveratrol released after 72 h at pH 7.4, reflecting controlled drug delivery. Biological evaluations included in vitro studies using neuroblastoma cells (SH-SY5Y) and human brain microvascular endothelial cells (HBMECs) to assess the ability of the nanoparticles to cross the BBB and effectively target neuronal cells. Results showed significantly enhanced uptake of Lf-RSV-PLGA-NPs, with fluorescence intensity in HBMECs reaching 8183.5 ± 1872.5 a.u. after 6 h compared to 3641.6 ± 1250.6 a.u. for non-functionalized nanoparticles, highlighting the importance of lactoferrin conjugation. Oxidative stress tests evaluated the antioxidant effects by measuring ROS levels in H_2_O_2_-exposed SH-SY5Y cells. Lf-RSV-PLGA-NPs reduced ROS levels to 592.5 ± 81.6 a.u. compared to 1696.8 ± 186.6 a.u. in untreated cells, demonstrating a significant neuroprotective effect. Mitochondrial membrane potential (MMP) assays further tested the ability of the prepared nanoparticles to mitigate mitochondrial dysfunction. The nanoparticles improved MMP, reflected by an aggregate/monomer fluorescence ratio of 6.3 ± 0.2 versus 3.7 ± 0.2 in untreated, 1-methyl-4-phenylpyridinium (MPP+)-damaged cells (MPP is a neurotoxic compound causing mitochondrial dysfunction and neuronal cell death). In vivo studies using C57BL/6 mice, treated with 1-methyl-4-phenyl-1,2,3,6-tetrahydropyridine (MPTP) to induce PD, validated the therapeutic efficacy of the nanoparticles administered intravenously. Restoration of striatal dopamine levels, measured using HPLC, reached 4265.6 ± 611.0 ng/g tissue in nanoparticle-treated mice compared to 2000.8 ± 111.4 ng/g in MPTP control mice. Behavioral tests demonstrated significant improvement in motor functions, with Lf-RSV-PLGA-NP-treated mice completing the beam test in 10.9 ± 4.0 s versus 24.2 ± 5.3 s in MPTP-mouse model and exhibiting more rearing behavior in Lf-RSV-PLGA-NP-treated mice compared to MPTP control (16 ± 3 times vs. 6.0 ± 2.5 times, respectively). To assess neuroinflammation, ICH analysis of glial activation showed reductions in astrocyte activation to 30.6 ± 2.6% and microglial activation to 25.7 ± 3.1%, compared to 40.8 ± 3.5% and 37.9 ± 4.3%, respectively, in control. Additionally, proinflammatory cytokines TNF-α and IL-1β were significantly reduced in the substantia nigra, with TNF-α/GAPDH and IL-1β/β-actin ratios lowered by 4.5-fold and 14.1-folds, respectively, compared to untreated MPTP-mouse model. Collectively, these results confirmed that Lf-RSV-PLGA-NPs effectively target the brain, reduce oxidative stress and neuroinflammation, and protect dopaminergic neurons, making them a promising therapeutic option for PD^131^.

**Fig. 12:**
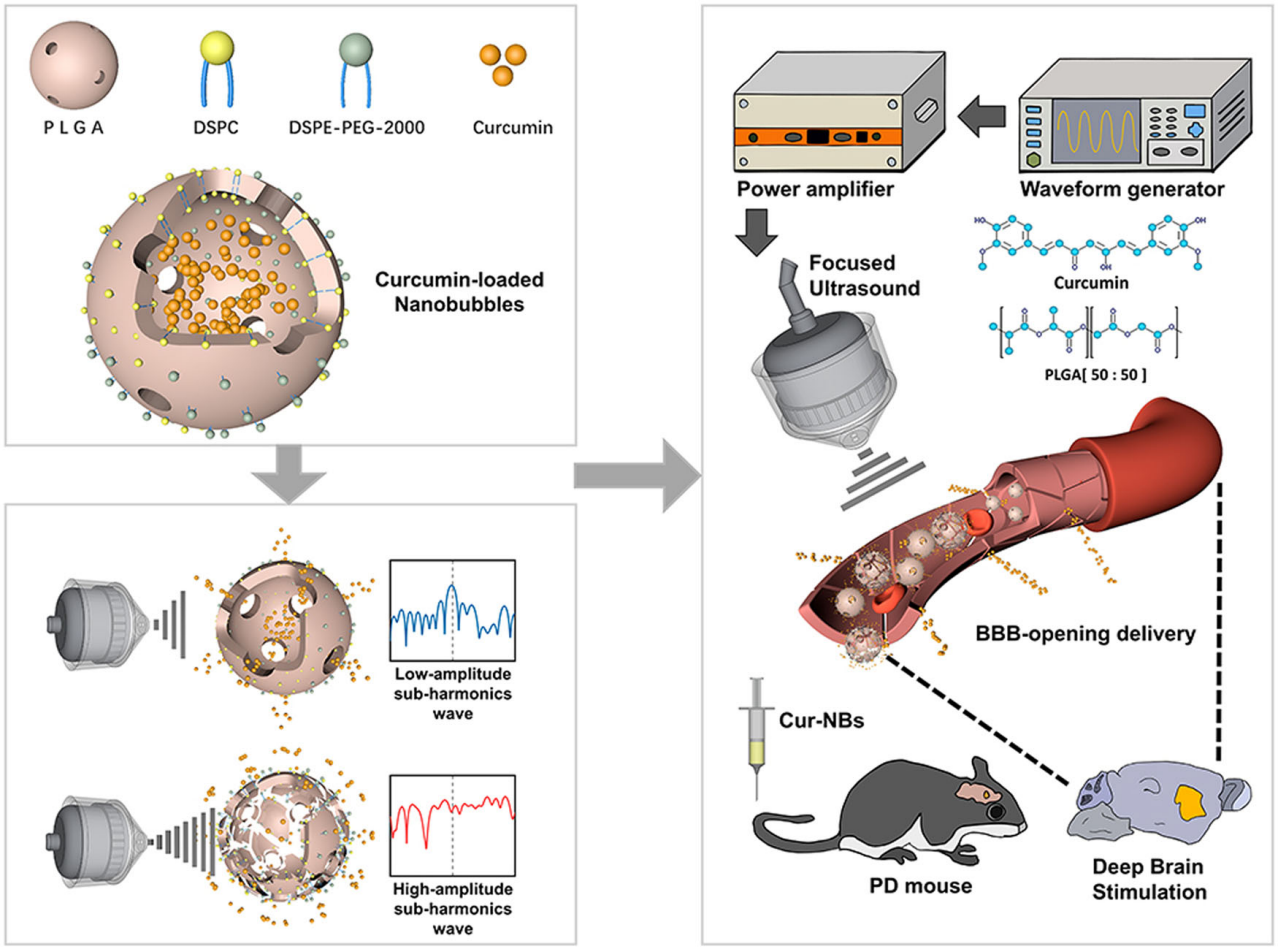
Schematic representation of Curcumin-PLGA nanoparticles designed for targeted drug delivery using LIFU-induced BBB opening via acoustic cavitation, and the delivery of curcumin to deep brain regions in a PD mouse model. This innovative approach enhances the bioavailability and therapeutic efficacy of curcumin for neurodegenerative diseases^132^. International Journal of Nanomedicine 2021, 16, 7433–7447. Originally published by and used with permission from Dove Medical Press Ltd.

Yan et al. developed PLGA nanosystem for the targeted delivery of curcumin in PD (Fig. 12)^132^. The nanosystem was designed to overcome the BBB using low-intensity focused ultrasound (LIFU) and improve poor bioavailability of curcumin. Curcumin-PLGA nanoparticles were fabricated using a double-emulsion solvent evaporation method after enhancing curcumin solubility through melt-crystallization, which dramatically increased curcumin solubility by 2627-fold compared to the pure compound. The Curcumin-PLGA nanoparticles exhibited an average size of 436 ± 58.3 nm, PDI of 0.31 ± 0.16, and a zeta potential of −27.5 ± 0.6 mV. Stability testing confirmed that the Curcumin-PLGA nanoparticles maintained consistent particle size and zeta potential for at least three weeks. In vivo biological tests utilized C57BL/6 mice subjected to a subacute PD model induced by daily intravenous administration of 1-Methyl-4-phenyl-1,2,3,6-tetrahydropyridine (MPTP) (40 mg/kg) for seven days. Curcumin-PLGA nanoparticles were administered intravenously every two days (6 doses in total), followed by LIFU-mediated BBB opening at an acoustic pressure of 0.45 MPa. Behavioral tests, including the rotarod test, assessed the motor coordination and neuromuscular function, and revealed significant improvements in the Curcumin-PLGA nanoparticles + LIFU group, compared to those treated with Curcumin-PLGA nanoparticles alone, LIFU alone, or untreated PD model mice. Similarly, the climbing test, which evaluates motor abilities, demonstrated enhanced performance in the Curcumin-PLGA nanoparticles + LIFU group, when compared to those treated with Curcumin-PLGA nanoparticles alone, LIFU alone, or untreated PD model mice. The hemolysis assay, performed to determine biocompatibility, showed negligible hemolysis rates (<5%) even at a high concentration of 1600 µg/mL of curcumin-PLGA nanoparticles, confirming their safety for intravenous use. Ultrasound imaging and BBB opening experiments validated the ability of LIFU-mediated Curcumin-PLGA nanoparticles to effectively deliver curcumin into deep brain regions. Curcumin penetration into the cerebral cortex and striatum was significantly enhanced, with delivery levels at 0.45 MPa acoustic pressure being 1.45-fold higher than at 0.31 MPa. These findings support the therapeutic promise of this innovative nanosystem for treating neurodegenerative disorders such as PD^132^.

#### Flavonoids

Dashputre et al. aimed to develop and evaluate a naringenin-loaded PLGA nanoparticulate for the targeted treatment of paraquat-induced PD rats^133^. Naringenin is a naturally occurring flavanone found in citrus fruits and figs, known for its antioxidant and neuroprotective properties, with the ability to cross the BBB. The nanoparticles were prepared using a single emulsion solvent evaporation method, exhibiting a particle size of 162.1 nm, a PDI of 0.288. The in vitro drug release profile achieved 54.62 ± 1.4% release over 10 h, indicating enhanced stability and bioavailability. For biological evaluation, male Wistar rats were employed for oral administration of the prepared nanoparticles at doses of 50 mg/kg and 100 mg/kg. Behavioral assessments included the rotarod test and open-field maze test. The rotarod test showed significant improvements in motor coordination, with the 100 mg/kg dose group demonstrating retention times nearly comparable to treatment with the standard PD drug bromocriptine (10 mg/kg). In the open-field test, line crossings and rearing behaviors were restored significantly in the 100 mg/kg group, indicating improved exploratory activity. Biochemical analyses revealed substantial reductions in oxidative stress markers. The malondialdehyde (MDA), a biomarker of oxidative stress, levels significantly decreased in the treated groups (*P* < 0.0001), with the 100 mg/kg group showing the lowest levels. Antioxidant enzyme activities, including SOD, catalase, and GSH, were significantly elevated in treated groups (*P* < 0.001). IHC revealed decreased α-synuclein protein levels, indicating reduced neuronal damage, and increased brain-derived neurotrophic factor (BDNF) levels, confirming neuroprotection. Histopathological evaluations corroborated these findings, showing reduced neuronal degeneration in the 100 mg/kg treatment group compared to the control group (treated only with paraquat to induce PD). These results highlight the potential of this nanosystem as a promising therapeutic option for PD^133^.

**Fig. 13:**
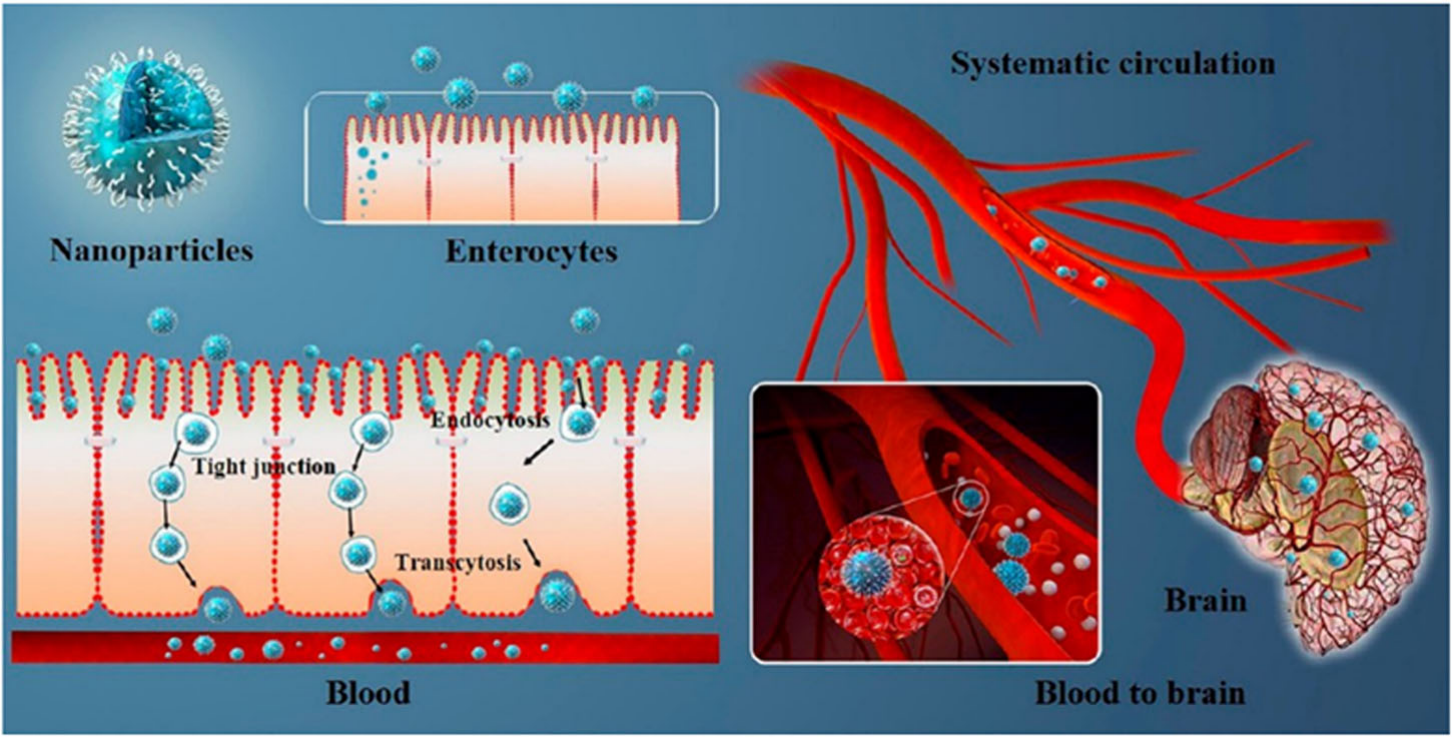
Diagram illustrating absorption of orally administered puerarin-loaded PLGA nanoparticles via intestinal enterocytes through endocytosis and transcytosis, overcoming tight junction barriers to enter systemic circulation. From the bloodstream, the nanoparticles efficiently cross the BBB to accumulate in the brain for PD treatment. Reprinted with permission from ref. ^134^, American Chemical Society, 2019.

Chen et al. developed a puerarin-loaded PLGA nanosystem aimed at enhancing the bioavailability and brain delivery of puerarin for the treatment of PD (Fig. 13)^134^. Puerarin is a flavonoid extracted from *Pueraria lobata* which showed neuroprotective effects in PD models. However, it has a number of limitations including poor water solubility, low bioavailability, and limited BBB penetration ability. The nanoparticles were prepared using an antisolvent precipitation method, producing spherical nanoparticles with an average size of 88.36 ± 1.67 nm, a PDI of 0.047 ± 0.007, and an encapsulation efficiency of 89.52 ± 1.74%, coupled with a sustained-release profile where 93% of puerarin released over 48 h. Biological evaluations included both in vitro and in vivo tests. In vitro studies using MDCK cells (a model of intestinal epithelial permeability) demonstrated that PLGA nanoparticles significantly enhanced cellular uptake and transport of puerarin across cell monolayers, achieving a permeability coefficient value of 2.83 ± 0.27 × 10^−5^ cm/s, which was significantly higher than that observed for free puerarin (0.38 ± 0.07 × 10^−5^ cm/s). In SH-SY5Y cells, the nanosystem showed superior cytoprotection against MPP+-induced cytotoxicity, increasing cell viability to 98.66% at 50 μM compared to 44% viability in untreated controls. Lactate dehydrogenase (LDH) release and ATP levels confirmed these protective effects, showing a reduction in LDH release from 212.29% to 122.98% and restoring mitochondrial membrane potential to 94.62% at 50 μM of the puerarin-loaded PLGA nanoparticles. Mitochondrial function, assessed by measuring oxygen consumption rate, demonstrated a 43.70% increase in basal respiration. Puerarin-loaded PLGA were orally administered to Sprague-Dawley rats, where pharmacokinetic analysis showed a 10.52-fold increase in plasma bioavailability and a 6.46-fold increase in brain puerarin accumulation compared to oral administration of free puerarin. Behavioral studies in a mouse model of MPTP-induced PD revealed that puerarin-loaded PLGA significantly reduced bradykinesia (pole test) and improved motor coordination (rotarod test), while restoring average travel distance and speed. Neuroprotective efficacy was evidenced by a 62% reduction in the loss of tyrosine hydroxylase+ neurons in the substantia nigra and substantial recovery of striatal dopamine and metabolites. Additionally, zebrafish embryos demonstrated no morphological abnormalities, toxicity, or changes in hatching, survival, or heart rates following exposure to puerarin-loaded PLGA nanopartilces^134^.

#### Coumarins

**Fig. 14:**
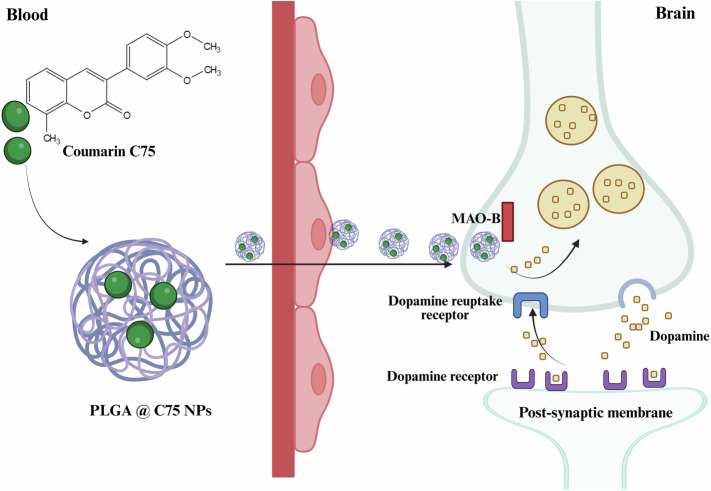
PEGylated PLGA nanoparticles encapsulating coumarin C75 for targeted inhibition of monoamine oxidase B (MAO-B) in PD. The nanoparticles enable efficient crossing of the BBB, enhancing dopamine levels at the post-synaptic membrane by reducing dopamine degradation, thereby addressing neurodegenerative challenges associated with PD. Redrawn with permission from ref. ^135^, American Chemical Society, 2018.

Fernandes et al. encapsulated coumarin in PEGylated PLGA nanoparticles to improve solubility, bioavailability, and delivery to the brain for the treatment of PD (Fig. 14)^135^. The nanoparticles were prepared using the nanoprecipitation method, resulting in a mean size of 105 nm with a low PDI 0.05 ± 0.01, indicating a uniform size distribution. The encapsulation efficiency was ~53.3%, achieving a therapeutic concentration 27,828 times higher than the IC50 value of free coumarin (28.89 nM). Controlled-release studies showed release of 45.8% coumarin over seven days, indicating sustained release. Biological tests were conducted both in vitro and in vivo. In vitro cytotoxicity assays using SH-SY5Y (human neuroblastoma), Caco-2 (intestinal permeability), and hCMEC/D3 (human brain endothelial) cells demonstrated that nanoencapsulation significantly reduced the inherent cytotoxicity of free coumarin. While free coumarin at 50 µM reduced cell viability below 82% in SH-SY5Y cells, the nanoencapsulated form showed no cytotoxic effects at this concentration. Permeability studies on Caco-2 and hCMEC/D3 cell monolayers showed enhanced intestinal and brain permeability of the encapsulated drug, with a 3.4-fold increase in coumarin transport across intestinal cells and a 25.6% increase in BBB permeability. Cellular uptake studies revealed a 5.3-fold increase in drug internalization in Caco-2 cells when encapsulated in nanoparticles. ATPase activity assays confirmed that coumarin stimulates ATP hydrolysis by P-glycoprotein (P-gp), indicating that coumarin acts as a substrate for it, and the nanoparticles were shown to inhibit P-gp-mediated efflux, enhancing drug retention in cells. Rhodamine 123 accumulation studies confirmed these findings, showing a 2.41-fold increase in intracellular retention of the dye when nanoparticles were used to deliver Rhodamine. In vivo studies using rats (oral administration) validated the enhanced pharmacokinetic profile of the nanoparticles. The encapsulated drug demonstrated prolonged circulation time and improved bioavailability, with sustained plasma concentrations compared to the free drug. These results highlight the potential of coumarin-loaded PEGylated PLGA nanoparticles as a robust drug delivery system for addressing bioavailability and permeability challenges associated with neurodegenerative disease treatments^135^.

## Clinical translation of PLGA-based nanoparticles in PD

Rotigotine is a dopamine agonist used in PD management. The incorporation of rotigotine into an extended-release microspheres (LY03003) was designed to overcome limitations associated with the existing rotigotine formulation, Neupro®, a daily transdermal patch. While Neupro® delivers continuous dopaminergic stimulation, its use is limited by the need for daily application and possible local skin reactions (erythema and contact dermatitis)^136^. Rotigotine encapsulated in PLGA microspheres allowed for once-weekly intramuscular administration^136^. The toxicity of intramuscularly administered rotigotine behenate-loaded PLGA microspheres (rotigotine loading of 22.5–27%) was evaluated over a 20-week period in Sprague Dawley (107 males and 107 females) rats which received 5 doses of rotigotine behenate-loaded PLGA every 28 days at different doses 60, 180, and 540 mg/kg (calculated as rotigotine)^137^. Toxicokinetic analysis showed a dose-dependent increase in plasma rotigotine concentrations. In comparison to the values observed after the first dose, the maximum plasma concentration (Cmax) increased by 3.97–4.88 times and the area under the curve (AUC_0_–_28_d) increased by 6.33–7.04 times. This level of accumulation was considered moderate showing sustained drug release over long dosing intervals. Treated rats developed white nodules at the injection sites. These local reactions were reversible at the end of recovery period (12 weeks after the final dose). Furthermore, hormonal analysis revealed a dose-dependent suppression of prolactin levels in female rats. This was attributed to the increase of ovary-to-body weight ratios and ovary-to-brain ratios along with mammary acinar hypertrophy and ductal epithelial hyperplasia. No abnormalities were observed in hematological or biochemical indices, urinalysis, or behavioral and ophthalmic assessments. All adverse effects were reversible during the recovery phase. The no observed adverse effect level (NOAEL) was determined to be 540 mg/kg, equivalent to 24 times the human clinical dose (448 mg/person/28 days), supporting the safety margin of rotigotine behenate loaded in PLGA microspheres and its potential for advancement into phase I clinical trials for PD^137^.

A similar study was conducted by Zhao et al. on cynomolgus monkeys (25 males and 25 females, aged 3–5 years) utilizing rotigotine behenate-loaded PLGA microspheres (rotigotine loading of 56.2–56.7%) as a long-acting dopamine agonist formulation for the treatment of PD through continuous dopaminergic stimulation^138^. Intramuscular injections of 90, 180, and 360 mg/kg doses were administered every 28 days for five doses, followed by a recovery period (12 weeks). Toxicokinetic analysis revealed that rotigotine behenate-PLGA exhibited sustained release, where Cmax and AUC_0_–_28_d increased proportionally with the dose with moderate accumulation of rotigotine. In hematological assessments, an increase in white blood cell, neutrophils, basophils, and fibrinogen count was found after the 3rd and 5th injections that were reversed to normal levels by the end of the recovery period. Likewise, serum prolactin levels were suppressed in a dose-dependent manner and monkeys developed gray-white nodules with granulomatous inflammation at the injection sites. Furthermore, clinical chemistry tests, urinalysis, bone marrow smears, neurological function tests, ophthalmic exams, and organ weight analyses were normal. Electrocardiogram, blood pressure, and respiratory rate remained stable, with only mild temperature elevations noted in male monkeys at all doses and females at 360 mg/kg. The study showed that the NOAEL of rotigotine behenate-loaded PLGA was at 360 mg/kg, supporting its safety and potential for clinical translation^138^.

The same research group investigated the fertility, genotoxicity, and embryonic development toxicity of rotigotine behenate-loaded PLGA^139^. NIH mice (30 males and 30 females) were utilized to conduct bone marrow micronucleus assay after single intramuscular doses of 167, 333, and 667 mg/kg. The study showed no observed increase in micronucleated polychromatic erythrocytes, confirming absence of chromosomal damage. For reproductive toxicity evaluation, Sprague Dawley rats (26 males and 26 females) received doses at 60, 180, or 540 mg/kg every 28 days (males on days 1, 29, and 57; females on days 1 and 29). Sperm analysis and mating performance showed no differences between rotigotine behenate-loaded PLGA and control males. However, female rats exhibited reduced fertility and pregnancy rates at all doses with uterine swelling, and increased corpora lutea counts. In pregnant females, no significant changes in organ weights or fetal outcomes were detected. The study concluded that rotigotine behenate-loaded PLGA did not induce genotoxicity but impaired female fertility, likely due to dopamine receptor-mediated suppression of prolactin^139^.

A population of human subjects with early-stage PD were recruited to investigate the pharmacokinetic properties of rotigotine-PLGA (LY03003)^136^. For this purpose, the study was conducted across China, Japan, and the United States, involving a total of 105 human participants (60 males and 45 females, 72.4% Asian and 27.6% non-Asian). The subjects received intramuscular injections of LY03003 (rotigotine loading of 22.5–27%) in an escalating dose regimen. The dosing schedule began at 14 mg in week 1, followed by 28 mg (week 2), 42 mg (week 3), and 56 mg for five consecutive weekly administrations (week 4, 5, 6, 7, 8). Blood sampling was performed following the first and fifth doses (week 1 and week 5), with blood samples collected at multiple time points to assess drug concentration. Results showed a biphasic plasma concentration–time profile, with an initial peak occurring within 24 h (initial absorption rate of 0.02 L/h) and a secondary sustained release phase (delayed absorption rate of 0.01 L/h) attributed to the gradual degradation of PLGA. The drug also distributes widely in the body (volume of distribution of 462,579 mL) and cleared at a rate that supports once-weekly administration (apparent clearance of 16,758 mL/h). Covariate analysis showed that body weight appeared to significantly influence clearance, while race influenced volume of distribution. However, other factors like age, body mass index, sex, albumin, liver enzymes, and creatinine clearance had no meaningful impact. Safety assessments indicated that LY03003 was well-tolerated, with the most common adverse events being induration at the injection site, dizziness, nausea, and orthostatic hypotension^136^.

Clinical trials have been established for the assessment of the pharmacokinetics, safety, and therapeutic potential of LY03003. In a Phase I crossover study involving healthy volunteers (NCT04384666), the pharmacokinetic profile of a single dose of LY03003 (28 mg) injected intramuscularly was compared with a 7-day regimen of Neupro®, indicating that the injectable system could provide sustained drug release over an extended period^140^. Another Phase I trial (NCT04629404) was conducted on early-stage PD patients to assess pharmacokinetics, pharmacodynamics, and safety of LY03003, demonstrating well tolerability and promising pharmacodynamic effects, suggesting its suitability for continuous dopaminergic stimulation^141^. Further extending the clinical evaluation, a Phase I trial (NCT04630860) focused on advanced-stage PD patients to assess the safety and pharmacokinetics of LY03003 via intramuscular administration. This study aimed to determine the appropriate dosing regimen for this patient population, ensuring both efficacy and tolerability^142^. Building upon these findings, a subsequent Phase III study (NCT04571164) was conducted to further evaluate the tolerability, safety, and pharmacokinetics of LY03003 in a larger cohort of PD patients, aiming to optimize dosing regimens and confirm its potential as a long-acting therapeutic option^143^.

## Challenges and future perspectives in the clinical translation of PLGA nanoparticles for PD

The previously discussed preclinical studies have shown the benefits of loading drugs and natural compounds into PLGA nanoparticles to enhance therapeutic efficacy, minimize systemic toxicity, and overcome peripheral side effects associated with PD medications. However, despite the experimental evidence, clinical translation of PLGA-based nanoparticles for PD remains limited. This could be attributed to the fact that the assessment of PLGA-based nanoparticles for PD treatment focused mainly on dopamine levels and short-term symptomatic improvements^117,121,124,126^. Another crucial challenge is the BBB penetration. PLGA nanoparticles have demonstrated ability to cross BBB through PEGylation, size/surface charge tuning, and receptor mediated targeting. However, achieving efficient drug delivery into substantia nigra region in the brain remains a fundamental challenge^144,145^. PD is an idiopathic neurodegenerative disorder characterized by heterogeneous pathophysiology with different molecular mechanisms across patients. Importantly, even the integrity of BBB can vary among PD patients, altering permeability of therapeutic agents and further complicating the development of effective PLGA nanoparticle therapies^145,146^.

Additionally, unmodified PLGA nanoparticles are cleared rapidly by the spleen and liver through the reticuloendothelial system (RES), shortening their circulation time and therapeutic window^99^. However, if modified (PEGylation or ligand-functionalization), PLGA nanoparticles could escape the RES clearance, allowing longer systemic plasma half-life and accumulation in the CNS^99^. Upon delivery to CNS, they degrade primarily via acid hydrolysis into their monomers (lactic and glycolic acids). The localized accumulation of degradation products in the brain lowers the pH, promoting oxidative stress and neuroinflammation. Thus, this raises concerns about the long-term safety of PLGA-based delivery systems for PD^97,107^.

Furthermore, most PLGA nanoparticles are synthesized utilizing solvent evaporation method, rendering it difficult to be reproduced at large scale under good manufacturing practice (GMP) conditions^110,147^. Minor inconsistencies in size, surface charge, or encapsulation efficiency can impact their therapeutic efficacy. Thus, transitioning from lab-scale to clinical-grade production requires standardization^147^.

Because PLGA-based nanoparticles have the potential to move from proof of concept and become a transformative solution in PD therapeutics, novel strategies to overcome their limitations are warranted. Future designs could further investigate the ligand-directed targeting strategies to enhance BBB penetration^29^. Additionally, bypassing the BBB through intranasal routes deserves further exploration. For effective PD treatment, symptomatic agents (levodopa) and disease-modifying compounds (antioxidants or anti-inflammatory agents) could be co-delivered to enhance the treatment outcomes^145^. Co-encapsulation of synthetic drugs and natural bioactive chemicals within the same PLGA nanoparticle system could have a synergetic therapeutic effect. Furthermore, technologies such as microfluidics or flash nanoprecipitation should be explored as they offer greater control over nanoparticle size and composition to ensure consistent biological response and GMP compliance^145,148,149^. Another priority is the establishment of preclinical models that better simulate the heterogeneity of PD, so PLGA-based nanoparticles could be tested under physiologically relevant conditions^150^. This could facilitate the progression of PLGA-based nanoparticles from laboratory-scale research to clinically viable therapeutics and eventual clinical trial implementation.

## Conclusions

PD remains a challenging neurodegenerative disease due to its complex pathology and limitations in existing therapies. While pharmacological treatments and phytochemicals could provide symptomatic relief and neuroprotective effects, their efficacy is hindered by poor bioavailability, systemic side effects, and restricted BBB penetration. Nanocarriers including PLGA-based nanoparticles emerge as a promising solution, offering enhanced drug stability, controlled-release, and targeted delivery. By encapsulating pharmacological agents and natural compounds, PLGA systems could overcome the shortcomings of conventional therapies and improve the therapeutic potential of phytochemicals.

Clinical translation of the developed PLGA-based nanosystems encapsulating conventional drugs and/or bioactive phytochemicals remains a challenge due to limited clinical trials validating the safety and efficacy of PLGA nano-systems. Complex manufacturing processes and the need for precise control over particle properties hinder scalability, while immune responses and inefficient BBB targeting present additional obstacles. The lack of multi-drug delivery platforms and long-term stability further restricts the clinical utility of PLGA nanosystems. Future efforts should focus on developing strategies to enable multi-drug encapsulation, enhance long-term stability, and personalize treatments based on patient-specific data. Finally, conducting large-scale clinical trials of the optimized nanosystems loaded with conventional PD drugs and/or phytochemicals would be essential.

## Data Availability

No datasets were generated or analyzed during the current study.
